# An Indicator Measuring the Influence of the Online Public Food Environment: An Analytical Framework and Case Study

**DOI:** 10.3389/fnut.2022.818374

**Published:** 2022-06-30

**Authors:** Na Cong, Ai Zhao, Mei-Po Kwan, Jun Yang, Peng Gong

**Affiliations:** ^1^Department of Earth System Science, Institute for Global Change Studies, Ministry of Education Ecological Field Station for East Asian Migratory Birds, Tsinghua University, Beijing, China; ^2^Vanke School of Public Health, Tsinghua University, Beijing, China; ^3^Department of Geography and Resource Management and Institute of Space and Earth Information Science, The Chinese University of Hong Kong, Shatin, Hong Kong SAR, China; ^4^Ministry of Education Ecological Field Station for East Asian Migratory Birds, Tsinghua University, Beijing, China; ^5^Department of Geography and Earth Sciences, The University of Hong Kong, Pok Fu Lam, Hong Kong SAR, China

**Keywords:** online public food environment, exposure, takeaway food, indicator/measurement, dietary diversity, analytical framework

## Abstract

The online public food environment (OPFE) has had a considerable impact on people's lifestyles over the past decade; however, research on its exposure is sparse. The results of the existing research on the impact of the food environment on human health are inconsistent. In response to the lack of food elements in the definition of the food environment and the lack of a clear method to assess the health attributes and the impact degree of the food environment, we proposed a new analytical framework based on the latest disease burden research, combining the characteristics of China's current food environment, from the perspective of environmental science. We redefined the food environment and proposed that food and its physical space are two core elements of the food environment. Accordingly, we extracted four domains of characteristics to describe the basic components of the food environment. Using the sales records, we designed an approach by referring to the standard process of environmental health indicators, including the health attributes and the impact degree of the food environment, to measure the OPFE of takeaway food outlets. Further, we conducted a case study and extracted three domains of characteristics for more than 18,000 effective takeaway meals from 812 takeaway food outlets located in 10 administrative subdivisions in the Haidian District and Xicheng District of Beijing Municipality. The results showed that more than 60% of single meals sold by takeaway food outlets were considered as healthy, and only 15% of takeaway food outlets sold healthy meals exclusively. Additionally, there were significant differences in health effects among different types of food environments, and high-risk areas of different types of food environments can be spatially identified. Compared with the counting method in the availability of food environment, the proposed new approach can depict food environment characteristics not only in the macro-scale like the counting method but also in the meal-scale. The indicators could be useful for large-scale and long-term monitoring of food environmental changes due to their simple calculation and design depending on the food delivery platform.

## Introduction

Currently, many countries have multiple forms of malnutrition, from the individual to the national scale (1). The Global Nutrition Report 2018 notes that about 88% of the 141 countries it analyzed have been experiencing more than one form of malnutrition, and 29% of countries have high levels of malnutrition in all three forms (2). In the form of malnutrition, the prevalence of overweight and obesity has increased in many developing countries over the past four decades (3). Among them, the prevalence of overweight and obesity in China is worrying. According to a recent official report, more than half of Chinese adults are overweight or obese (4). Obesity is not only a disease, but also a symptom (5) and cause (6, 7) of many chronic diseases. Previous practical intervention, such as educational, behavioral, and pharmacological (8), have been ineffective, as no country has successfully prevented the obesity pandemic (9). Additionally, undernutrition is also a pressing concern, especially in the low-and middle-income countries (LMICs) (10). Although the undernutrition- and micronutrient malnutrition-related health problems have been mitigated in China (4), these forms of malnutrition interweaving with overweight and obesity (10) make further prevention of malnutrition more difficult. As one of the many same underlying causes of malnutrition, the food environment has become a studied object to explore environmental interventions to mitigate malnutrition.

In its original definition, food environments comprise all “collective physical, economic, policy and socio-cultural surroundings, opportunities and conditions that influence people's food and beverage choices and nutritional status, such as food composition, food labeling, food promotion, food prices, food provision in schools and other settings, food availability and trade policies affecting food availability, price and quality” [(11), p. 8]. This definition broadly delimited the boundary of food environment but did not pinpoint the relationships among all components, and some food features, such as nutrition were considered beyond the scope of food environment research (12). Although a study reorganized two domains of the food environment measurements (13), parts of dimensions in the personal domain, such as desirability, were out of the scope of the food environment (14). Many studies in high-income countries have examined different dimensions of food environment measurement, within different conceptual frameworks (13, 15–18). However, no unified framework of measurement of the food environment has emerged. A recent study summarized eight measurement dimensions for low- and middle-income countries—accessibility, affordability, desirability, convenience, availability, price, product characteristics, and promotion/marketing. Of these, only four (availability, accessibility, affordability, and price) maintain consistency across frameworks (14). The availability and accessibility dimensions are the most frequently studied (19). In these two measurements, the characterization of the physical food environment includes different types of food outlets, such as fast-food outlets, supermarkets, grocery stores, etc., which are defined directly as healthy or unhealthy. A recent study evaluated the differences of characteristics from food supplied by different retail food outlets, such as supermarkets, grocery stores, and convenience stores across different types of neighborhoods (20); thus, we focus on the fast-food outlets in this study.

Most studies on the impact of a fast-food environment on human health proposed a latent hypothesis: that fast-food outlets are unhealthy because they sell energy-dense foods and drinks (21), especially in Europe and America. However, a recent study compared the food provided by different food outlets and found that not all fast-foods were unhealthy (22). In two other analyses, many other types of food outlets provided fast-food, including supermarkets and grocery stores, which are typically considered healthy food outlets (23, 24). Further, another latent hypothesis is that most studies considered the impact of food outlets to be the same on human health, except one study that weighted the food environment based on the rating of the experts (25). This hypothesis cannot reflect reality and therefore has been questioned in a recent study (26). Another study discussed how the error caused by this hypothesis impacts the result from the association model of food access and human health (27). All these issues might cause discrepant conclusions among studies on the association between the food environment and human health. Therefore, Cobb et al. (28) suggested the exploration of additional measurements.

While, research on the food environment in China is still in its early stages, several studies on the impact of the neighborhood food environment on people's diet and obesity have been conducted but with limited results (29). Thus, the abovementioned issues cannot be solved in China owing to the lack of a systematic framework and standard tools (30). The way to acquire food in China is changing tremendously. Currently, takeaway is the most popular form of food in China. The trend has been driven by the development of mobile internet and increasingly efficient delivery systems over the past decade (31). Although this has made the food environment in China more complex (32), the cumulative internet catering big data have also created better opportunities to explore human dietary behavior and characteristics of the food environment. The food delivery platform (FDP) is a potential tool for monitoring the food environment and mitigating the overweight and obesity concerns in China for four reasons. First, takeaway food outlets provide cooked food products — they can be consumed immediately upon reception; every single package may represent the individual's total potential food consumption per meal (food order). These food orders are recorded by an FDP (31), along with other food-related attributes, such as price, promotions, packaging fees, distribution fees, and the type and weight of ingredients. Second, FDPs cover most cities in China and have accumulated data in metropolitan areas for nearly 10 years. The broad spatial and longitudinal monitoring of food orders provides an unprecedented amount of data for food environment research (33). Third, the cost of data collection is lower than that of other types of food environment constructs (Table 1). Finally, FDPs have had a considerable impact on contemporary lifestyles. An important character is that the convenience of FDPs can save more time and make consumers' time-use more diverse. A qualitative study from Guangzhou found that at least 2 h a day could be “saved” by using FDPs (34). However, FDPs potentially promote a sedentary lifestyle and the consumption of unhealthy food (35), which is harmful to one's health, and food waste and packaging are harmful to the environment. Thus, to guide the change of the food environment toward a healthy and sustainable direction, FDPs will inevitably become the main intervention area to promote human health and environmental sustainability (31).

**Table 1:** Comparison of characteristics of different types of food outlets.

**Type of food outlets**	**Type of food provided**	**Who cooked the food?**	**How to know what kind of food they had in a meal**	**Cost of collecting the longitudinal data**
Online restaurants	Cooked	Chefs in the restaurants	Online purchasing record	Low
Offline restaurants	Cooked	Chefs in the restaurants	Survey	High
Online markets	Fresh or preprocessed	Consumers themselves or their family members	Survey	High
Offline markets	Fresh or preprocessed	Consumers themselves or their family members	Survey	High

With the above context, this study designed a new indicator to measure the online public food environment for takeaway food (OPFE-TF) and developed an approach to define the healthiness of food outlets and their various degrees of impact. Therefore, the indicators developed in this study were mainly used to monitor the characteristics of OPFE-TF, recognize the high unhealthy food risk areas, and prepare to model the associations between OPFE-TF and human health.

Specifically, we explored the following research questions: (1) How different are the healthiness and nutrition of food in different fast-food outlets? (2) How different is the health impact of different fast-food outlets? Considering the indicator measuring the OPFE-TF is an environmental health indicator (EHI), it was designed according to standard steps in the construction of EHIs (36). Supplementary Figure 1 shows the coupling of the structure of this study and the construction steps of EHI. In Section 2 Analytical framework and measurement of OPFE-TF, we defined the exposure-effect relationship between the food environment and human health, target point in Driving force-Pressure-State-Exposure-Effect-Action (DPSEEA) chain (37), and parameters on which the indicator will be based. Further, we prepared the data to test the indicator, evaluated its performance, and tried to answer the two research questions in Sections 3 and 4 Analytical framework and measurement of OPFE-TF and Case study.

## Analytical Framework and Measurement of OPFE-TF

### The Relationship Between Food Environment and Populations

**Figure 1:**
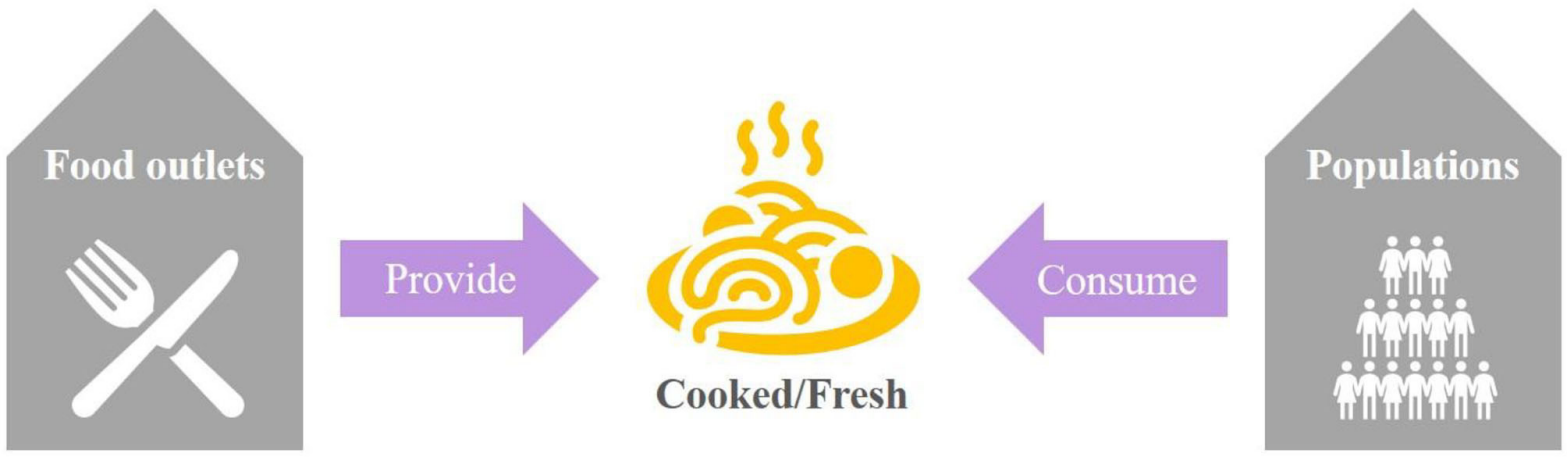
The fundamental fact of the relationship between food environment and populations.

In a systematic analysis about the global burden of disease, many risk factors for attributable deaths were correlated to food, such as dietary risk, high systolic blood pressure, high fasting plasma glucose, high low-density lipoprotein cholesterol, and child and maternal malnutrition, and so on (38). The food environment includes the main physical places for food storage and distribution in the food system (17), and it impacts human health in the process of interacting with people through food. Figure 1 shows that the food supplied by the food outlets is consumed by customers, making food the key element connecting people and food environment. Hence, it can be considered the core path through which the food environment affects people's health. Based on this relationship between the food environment and populations, food could be considered as the boundary of the food environment and connect the food environment and human population. The food environment can include the food attributes but not personal domains, such as desires, tastes, or attitudes. Thus, the characteristics of the food environment should be depicted by food attributes when we discuss the relationship between food environment and human health, and it should be consistent with the method to assess human dietary quality in nutrition science. All subsequent design of food environmental indicators would adhere to this principle.

### The Analytical Framework for OPFE-TF

**Figure 2:**
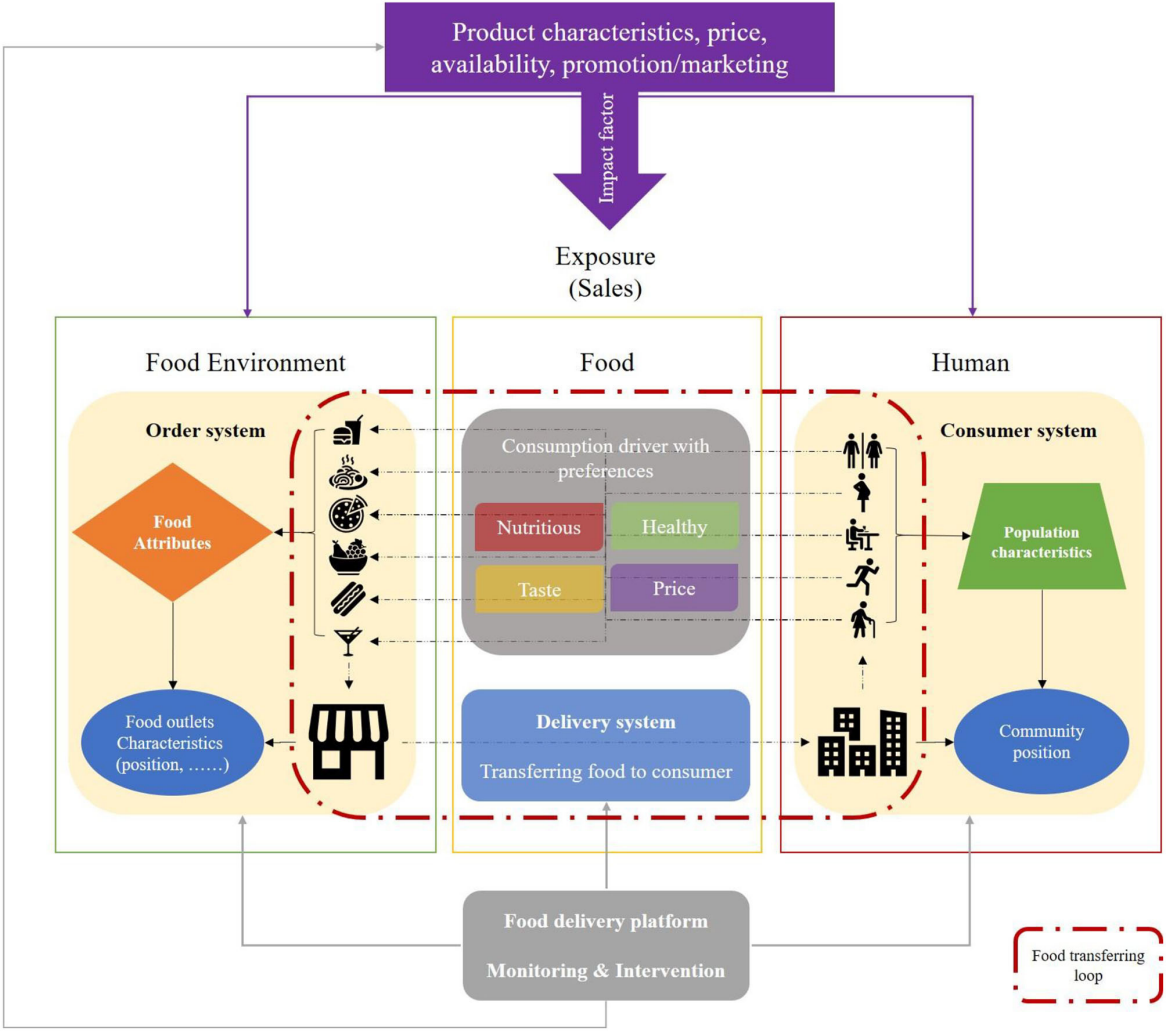
An analytical framework of the online public food environment.

According to the relationship between the food environment and populations, food could be considered as the boundary of the food environment linking the food environment and human population. This indicated that the food environment can include the food attributes, but not personal domains, such as desires, tastes, or attitudes. Based on this relationship, we constructed the analytical framework for OPFE-TF (Figure 2). The framework mapped the corresponding relationship between food environment-food-people and each operating system of the delivery platform. It illustrated how food connected the food environment and people during the transfer process between them. Following the analytical framework and the above principle, we proposed a new food environment definition:

*The food environment is a new type of environment composed of the food and the physical space carrying the food, as the interface between humans and the broader food system, interacting with the covered population constantly through economic, social, and cultural factors and influencing human health*.

This is a systematic definition from an environmental perspective. In the new definition, there are two key elements of the food environment: the food and the physical space carrying it. Therefore, the measurement of the food environment is separated into two levels. At each level, the dimensions describing the element of the food environment should be consistent. The only physical space that cannot be a food environment is one without food in it. Thus, the food is the precondition, and food attributes are fundamental to depict food environment characteristics.

The dietary risk factors identified in the global burden of disease research included 15 level III factors: diet low in fruits, diet low in vegetables, diet low in legumes, diet low in whole grains, diet low in nuts and seeds, diet low in milk, diet high in red meat, diet high in processed meat, diet high in sugar-sweetened beverages, diet low in fiber, diet low in calcium, diet low in seafood omega-3 fatty acids, diet low in polyunsaturated fatty acids, diet high in trans fatty acids, and diet high in sodium (7, 38–40). However, in reality, food impacts human health through steady accumulation, one meal at a time. Furthermore, most takeaway food on FDPs is sold in the form of one meal or a group of meals. Considering the single meal as the basic unit, we designed the indicator based on this unit in the environmental scale.

Among the dietary risk factors, trans fatty acids, and sodium typically come from oil and salt and are used as condiments in cooking; sugar is also a popular ingredient used in sugar-sweetened beverages, which may aggravate the excessive intake of energy. Therefore, we used oil, salt, and sugar to describe the health attributes of food. Fruits, vegetables, legumes, whole grains, and the rest of the factors are associated with food groups, and different combinations of foods from these food groups can form different types of meals. Quantitative calculation of food groups can evaluate dietary quality, a nutrition attribute. Therefore, food groups and their combinations can be used to describe the categories and nutritional characteristics of the food environment. Further, food prices, an important social and economic indicator, also affect the interactions between people and the food environment (13); therefore, price should also be a dimension to measure the food environment.

**Figure 3:**
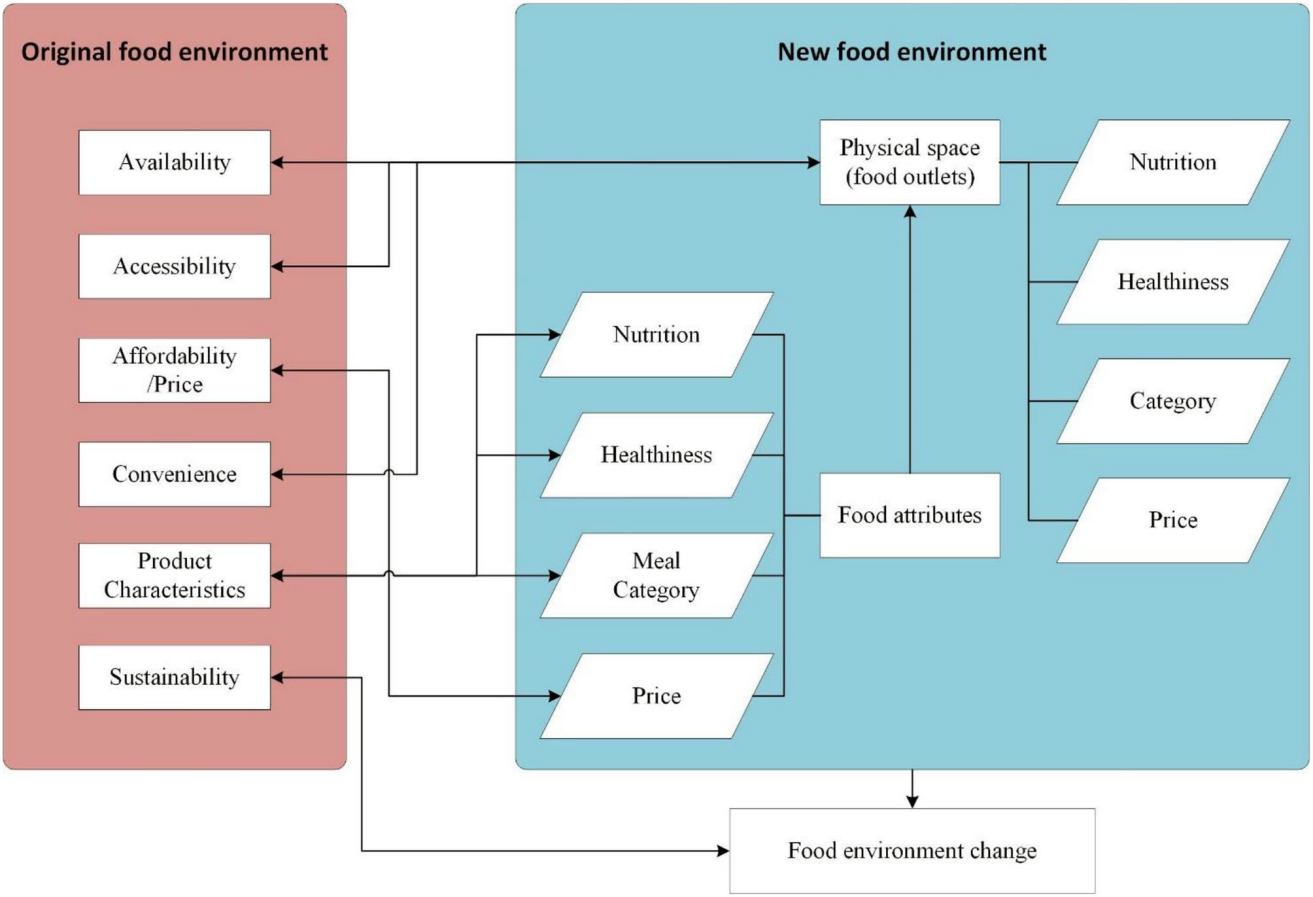
The transformational relationship among dimensions of the original food environment and the new food environment.

In sum, characteristics of food elements included four dimensions: nutrition, healthiness, category, and price, which are the factors considered by people most when choosing food in the analytical framework. To clarify the differences and similarities between the traditional food environment and the new one, the corresponding relationships among these measurement dimensions are mapped in Figure 3. In these four dimensions, price is a well-defined socioeconomic indicator; however, it is not suitable for depicting the physical characteristics of the food environment. Thus, we only designed the measurement of the other three dimensions.

The indicator proposed in this study was mainly used to identify unhealthy food outlets with high impact degree. Thus, the target point of this indicator was the state of the environment in the DPSEEA framework chain (37).

### Parameters of the Indicator

#### Category

In various studies (41–45), many food outlets were tagged by the Standard Industrial Classification or the North American Industry Classification System (NAICS) (46) or by researchers themselves (47, 48). However, these classification systems categorize economic entities based on their economic activities but not on the nutritional characteristics of their sold food. Take the “Restaurants and Other Eating Places^1^” in NAICS for instance, the classification of the other three categories is mainly based on the degree of service provided except for snacks and non-alcoholic beverages. In full-service and limited-service catering enterprises, some food outlets may serve similar food, such as pizzerias and steakhouses. Nevertheless, according to the basic rules of taxonomy (49), the NAICS classification system cannot effectively distinguish the characteristics of food served by outlets. The Industrial Classification for National Economic Activities in China (50) has the same problem. Therefore, a new classification system is necessary for fast-food outlets.

The balanced diet plate in the 2016 edition of the Dietary Guidelines for Chinese residents is a simplified way to describe the food composition and approximate proportion of a person's meal in accordance with the principle of a balanced diet without considering cooking oil and salt. As oil, salt, and sugar have been separated as a dimension to describe health attributes of food environment in the above definition, the balanced diet plate of Chinese residents can be used as a reference for the classification. Along with a previous food classification method (51) and the popular meal categories, a classification system for fast-food outlets and meals was proposed referring to the prior method of dietary patterns (Table 2). A two-level classification system was developed containing 3 Level I (Meal, Snacks and Beverage, and Other) and 14 Level II categories with corresponding Code I and Code II.

**Table 2:** The classification system for food outlets and meals.

**Level I**	**Code I**	**Level II**	**Code II**	**Description**	**Examples**	**Short name**
Meal	1	Staple	1-1	Rice is the major component in this type of meal with some meat and eggs	Rice soup, fried rice, etc.	ST
		Noodles and dumplings	1–2	Wheat and rice are the main components with some meat and vegetables	All kinds of noodles and dumplings	ND
		Set meal	1–3	Rice, meat/egg, and vegetables are the main components of this kind of meal. Sometimes there is some food from other food groups	/	SET
		Seafood	1–4	Seafood, fish and other aquatic products are the main components in this type of meal, with a small amount of vegetables and grains.	Paella, crayfish and rice, etc.	SF
		Pot	1–5	Vegetables, soy products, and aquatic products are the main ingredients	Hotpot, spicy hot pot, dry pot	POT
		Fried and BBQ	1–6	Meat is the main food and is cooked, fried, or smoked	/	F_BBQ
		Western fast-food	1–7	This includes western fast-food	Hamburger, pizza	WFF
		Healthy and light recipes	1–8	This includes average food from 80% of the food groups	/	HLR
Snacks and Beverage	2	Dessert	2-1	Snacks and beverages are sold separately	Cakes, doughnut, ice cream, etc.	DT
		Snacks	2-2		Non-ready-to-eat chicken wings, chicken legs, independently packed nuts, popcorn, all kinds of grilled sausage, etc.	SK
		Alcoholic beverage	2-3		Beer, white wine, wine, rice wine, sake, and other drinks containing a certain amount of alcohol.	AB
		Synthetic beverage	2–4		Cola, Sprite, and other carbonated drinks; fruit drinks; and other synthetic drinks.	SB
		Stimulating drinks	2–5		Red Bull and other functional drinks, tea, coffee, etc.	SD
Other	3	Unknown	3-1	This includes any food and food outlets outside of the above categories	/	UN

*The unknown category is not completely applicable for food. If a new food category is found, the classification system should be revised. Identification of unknown types in food should be minimized in later data processing*.

In the Meal category, there were eight Level II categories, in which many foods were set by food outlets in advance, such as staple foods, set meals, noodles and dumplings, western fast-food, and healthy light recipes. These types of meals can be easily recognized in a single meal. However, most pot meals (POT) and fried and barbecue meals (F_BBQ) were selected by consumer, and the seafood (SF) was sold by weight. Therefore, it is difficult to recognize these three types of meal in a single meal. One possible solution is to determine the category from the consumer side, because the order could be viewed as the basic unit of sales.

Moreover, the unknown type in the Other category was mainly used to describe features of the food outlet, not the food. The classification system should be revised if there are meals that do not belong to any existing categories. Additionally, western fast-food rushed in the catering market after the Chinese reform and opening-up. Their standard food processing and cooking mode were learned by other Chinese catering enterprises, which boosted the western fast-food supply considerably. The western fast-food was set to compare the differences of characteristics between Chinese and western fast-food and to explore the different impacts of them on Chinese citizens' health in the future.

##### The Rules for Categorizing Meals and Food Outlets

###### Rules for Classification of Meals

- When a meal is composited by a single Level II food in the classification system, this type of meal corresponds to Level II type.
- When a meal is composited by same or similar quantities of multiple Level I food, the type of the meal is decided by the priority of the category, which is decided by its code. Code 1 represents the highest priority. Subsequently, the food category is defined according to the number of Level II meal items.
- When a meal is composed of multiple Level I food items in different quantities, the food category is defined according to the most of Level II food items.

###### Rules for the Classification of Food Outlets

- Similar to the meal classification rules, when a food outlet sells only one meal type, it will be defined by the meal category.
- When the food outlet sells various meal types, the type of the food outlet is defined by the meal types it sells most.
- When the food outlet sells the same or a similar number of meal types, the type of the food outlet will be defined as the Unknown.

#### Healthiness

In the definition of the food environment, oil, salt, and sugar were grouped to describe the healthiness attribute of the food and food outlets. There are limitation standards for oil, salt, and sugar in most dietary guides across countries (52–55); thus, the healthiness of the meal can be ideally determined based on whether the weight of the oil, salt, or sugar exceeds the limitation standards. Pragmatically, however, it is difficult to obtain the actual content of oil, salt, and sugar in a meal, especially for takeaway food. Moreover, the intake standard of oil, salt, and sugar required in the dietary guidelines is usually calculated based on a person's daily or weekly intake. No study has discussed the intake standard of oil, salt, and sugar in a single meal. Therefore, the measurement of the healthiness of the meal is mainly characterized by a proxy method at present:


(1) $$\begin{array}{l} l=\sum j \end{array}$$

Here,

*l* is the healthy score of a meal, and *j* is a separate health label, calculated as follows:


(2) $$\begin{array}{l} j=\{\begin{array}{c} \begin{array}{c} 0,\text{ if neither of fried food},\text{ sweetened sugar beverage},\\ \text{or sauces or pickles with high salt in meals} \end{array}\\ \begin{array}{c} 1,\text{ if any one of fried food},\text{ sweetened sugar beverage},\\ \text{or sauces or pickles with high salt in meals} \end{array} \end{array} \end{array}$$

The maximum value of *l* is 3, indicating that all of fried food, sweetened sugar beverage, or sauces or pickles with high salt are in a meal, it is the unhealthiest meal. The minimum value of *l* is 0, implying that neither of fried food, sweetened sugar beverage, or sauces or pickles with high salt in meal, it is the healthiest meal.

#### Nutrition

The nutrition dimension was designed based on the principle proposed in the exposure-effect relationship between the food environment and human health. Nutrition is related to people's dietary quality. There are many individual dietary assessment methods in nutrition science, such as dietary diversity score (DDS) (56), healthy eating index (57), and diet quality index (58). Except DDS, most of the other dietary assessment methods require at least two of the following three key variables: food groups, intakes, and referenced intakes. However, for the OPFE-TF, it is difficult to obtain the weight of the raw food. Owing to the consideration of the proportion of different food groups in a meal in the category dimension along with its simple calculation, which is suitable to assess the nutrition characteristic of OPFE-TF, the DDS became a prime candidate. Furthermore, there are three reasons for choosing DDS as an indicator construct. First, DDS is designed by the Food and Agriculture Organization to assess whether an individual or family's food intake is adequately nutritious (56). In practical application, although there is some controversy concerning the association between DDS and population health (59, 60), it is still a useful indicator of overall diet quality, especially in large-scale surveys (60). Second, DDS characterizes the nutritional properties of food, and provides an opportunity to assess the sustainability of the food environment as a link to the ecosystem (61). Finally, the data for calculating DDS are easier to obtain from the FDPs compared with other nutritional indicators. Although food outlets on FDPs have not yet fully labeled the type and weight of their food (31), this shortcoming can be easily overcome by using incentives.

In sum, the DDS is the most suitable indicator to assess the nutrition dimension of the food environment till now. DDS is calculated as follows:


(3) $$\begin{array}{l} DDS=\;\overset{m}{\underset{k=0}{\sum }}FG_{k} \end{array}$$

Here, *DDS* is the dietary diversity score of a meal in the food outlet, and *k* is the index of food groups in a meal. Further, *FG* is the food group in a meal, and *m* is the total number of food groups in a meal.

#### Indicators for Measuring OPFE-TF

Among the three parameters, category is mainly used for the qualitative description of the food environment, and the healthiness and nutrition parameters are mainly used for quantitative measurements. For the indicator to clearly express a healthy or unhealthy food environment with a high impact, the actual meanings of these parameters should be analyzed in depth.

For the healthy score of a meal, a higher value indicates an unhealthier meal. A change in the direction of this parameter is consistent with that of an unhealthy food environmental indicator. When the value of the healthy score of a meal is 0, the meal is healthy. DDS is an indicator used to evaluate the overall dietary diversity of the population. A larger DDS value indicates abundant nutrition of the meal. A smaller DDS value indicates a poor nutritional profile of the meal. Thus, the parameter change direction of DDS is opposite to the unhealthy food environmental indicators. Therefore, for an unhealthy food environment, a transformation of DDS was necessary. As the original value of DDS is a non-zero, non-negative integer, and DDS is meaningless only when the food does not exist, a reciprocal transformation for DDS is conducted to change the direction of DDS value consistent with other parameters.

Sales is the accumulative total food consumption in a period. For OPFE-TP, the sale of a meal is recorded for 1 month. The higher the value of the sale, the more the meal is consumed, indicating that the meal in this food outlet has a strong impact on the population. However, sales have no clear impact direction. The healthy score and DDS are used to determine the healthiness and nutrition direction to construct the following indicator: Total unhealthy impact degree of the food environment (TUHII). It is calculated as follows:


(4) $$\begin{array}{l} \text{TUHII}=\;\overset{n}{\underset{i=1}{\sum }}l_{i}\;*\;\bar{\frac{1}{DDS_{i}}}\;*\;S_{i} \end{array}$$

Here, *i* is the index of the meal in a food outlet, and *n* is the total number of meals in a food outlet. Further, *l*_*i*_ is the healthy score of the *i* meal, *DDS*_*i*_ is the dietary diversity score of the *i* meal, and *S*_*i*_ is the sale in a month for the *i* meal. Additionally, $\bar{\frac{1}{DDS_{i}}}$ is the normalized reciprocal of *DDS*_*i*_.

Equation (4) measures the total unhealthy impact degree of food environment. The higher the value of TUHII, the stronger is the impact of unhealthy food outlets. However, when *l*_*i*_ is equal to 0, there will be no impact of healthy food outlets in the total food environment. With the increasing health awareness of Chinese citizens, healthy food outlets generated a more positive high impact on human health. The modeling on the association between the food environment and human health would involve more confounders if the impact of the healthy food outlets cannot be considered. Therefore, the result of the modeling would be inconsistent and invalid in supporting the designing of intervention strategies. Additionally, healthy food outlets should be fully considered when the food environment is expected to benefit human health. The impact of a healthy and unhealthy food environment is also expected to be compared horizontally. Hence, we divided food outlets into two groups by the health weight, which is calculated as follows:


(5) $$\begin{array}{l} W=\frac{N_{h}}{N_{t}} \end{array}$$

Here, W is the health weight of the food outlet, *N*_*h*_ is the total number of healthy meals, and *N*_*t*_ is the total number of meals.

The value of W is in 0–1. A food outlet with health weight 1 sold only healthy meals, while all meals sold in a food outlet with 0 health weight are unhealthy. Specifically, every meal sold in this food outlet included at least one unhealthy food. When the health weight is more than 0 and <1, the food outlet sold the healthy and unhealthy meals simultaneously. The higher the W is, the healthier meals are sold in this food outlet.

According to W and the meaning of DDS and the reciprocal of DDS, we divided the food outlets into two groups and separately constructed the impact degree for healthy food environment (HII) and unhealthy food environment (UHII), which are calculated as follows:

When W is equal to 1,


(6) $$\begin{array}{l} \text{HII}=\;\overset{n}{\underset{i=1}{\sum }}\bar{DDS_{i}}\;*\;S_{i} \end{array}$$

When W is less than 1,


(7) $$\begin{array}{l} \text{UHII }=\;\overset{n}{\underset{i=1}{\sum }}\;\bar{\frac{1}{DDS_{i}}}\;*\;S_{i} \end{array}$$

Here, *i* and *n* are the index and total number of meals in a food outlet, respectively. Further, *DDS*_*i*_ is the dietary diversity score of the *i* meal, and *S*_*i*_ is the sale in a month for the *i* meal. Additionally, $\bar{\frac{1}{DDS_{i}}}$ is the normalized reciprocal of *DDS*_*i*_.

In Equation (6), a higher value of HII corresponds to a stronger impact of the healthy food outlet. In Equation (7), a higher value of UHII, corresponds to a stronger impact of the unhealthy food outlet.

### Other Steps of EHI

For the statistical analysis, there are two ways to assess the food environment: (1) calculate the cumulative impact of all food outlets within a certain area, or (2) calculate the number of food outlets with the strongest unhealthy impact within a certain area. The first is the basic analysis method used in this study. There are many ways to aggregate this indicator in geography, such as the administrative areas, unit area or population, and buffered area for the food environment itself. We used administrative and unit areas to evaluate the differences of the impact degree of food environment geographically. To the best of our knowledge, this study is the first to discuss the fast-food environment assessment in China; therefore, there were no baseline data for reference. According to the method of geographic aggregation, maps are more suitable to express the result of the food environment assessment.

## Case Study

### Data Description and Processing

The Meituan Group is the largest FDP in China, with a market share of 68.2% in the second quarter of 2020 (62). The food outlets registered on this platform were collected by crawler technology in November 2020, yielding 42,002 food outlets, and a number of associated meals in Beijing. According to the Business Information Database of RESSET^2^ Enterprise Big Data Platform, there were approximately 64,000 catering enterprises in Beijing by the end of December 2020. The food outlet data we collected, covered most of the online food services in Beijing apart from the professional food services at the airport, schools, and some hotels providing only offline services. The data of food outlets only selling snacks and beverages cannot be collected owing to feasibility and other limitations. Thus, we only tested the food environment constructed by food outlets in the meal category.

**Figure 4:**
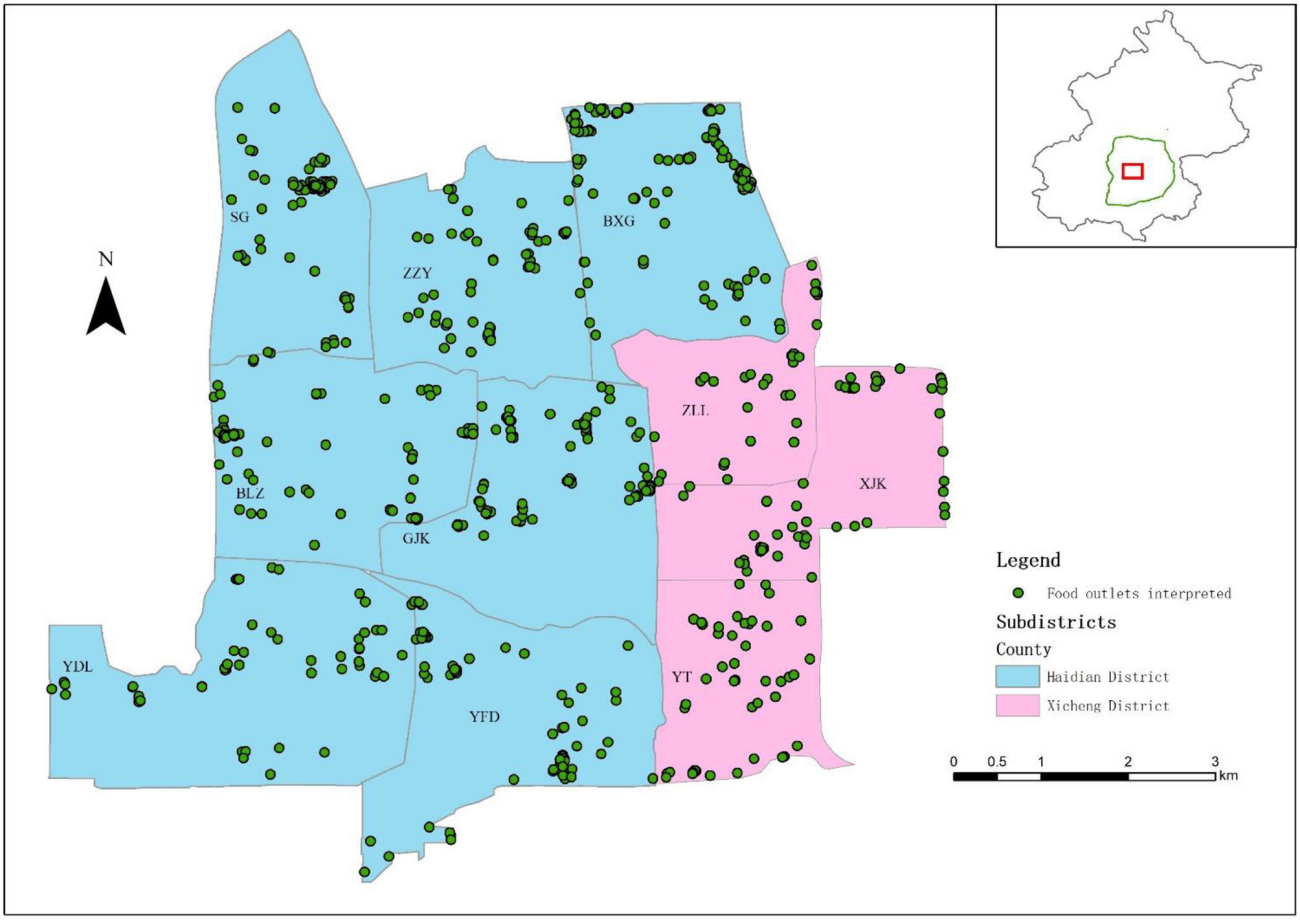
The study area and the spatial distribution of interpreted food outlets. BLZ, Balizhuang; BXG, Beixiaguan; GJK, Ganjiakou; SG, Shuguang; XJK, Xinjiekou; YFD, Yangfangdian; YDL, Yongdinglu; YT, Yuetan; ZLL, Zhanlanlu; ZZY, Zizhuyuan.

Most fast-food outlets located in the central urban area and the characteristics of the food environment are a long-term cumulative result of the interaction between the food outlets and consumers. Thus, 10 subdistricts were selected from the Haidian and Xicheng districts in Beijing: Balizhuang (BLZ), Beixiaguan (BXG), Ganjiakou (GJK), Shuguang (SG), Yongdinglu (YDL), Yangfangdian (YFD), Zizhuyuan (ZZY), Xinjiekou (XJK), Yuetan (YT), and Zhanlanlu (ZLL). The study area is shown in Figure 4. After spatial linking, more than 2,000 food outlets were selected in these 10 subdistricts. Details of the data processing are mapped in Supplementary Figure 2. As we constructed the indicator in the unit of one meal, we excluded the records of more than one meal and other items: (i) sauce ingredients; (ii) separate beverages or other drinks; (iii) concomitant food, such as that which cannot be delivered if it is solely ordered, or some packaged food; (iv) foods in the POT, fried and BBQ, or seafood categories; (v) set meals for multiple persons or group meals; (vi) separate stir-fried dishes, cold dishes, or separate staple foods (rice, steamed bread, etc.) that can be selected by consumers independently; (vii) items sold separately by delicatessen; (viii) single soups; (ix) other meals not in the Meal category, such as cakes, candies, dried fruits, and other snacks; and (x) other foods that cannot be recognized as a meal. After screening, nearly 20,000 meal samples from 847 food outlets were selected for interpreting and extracting food features.

The food group used to calculate DDS is divided into 12 groups referring to Chinese research (63) and food composition tables (64, 65): (1) cereals; (2) roots and tubers; (3) vegetables; (4) mushroom and seafood (plant); (5) meat, poultry, and offal; (6) eggs; (7) fish and seafood; (8) pulses and legumes; (9) nuts; (10) dairy products; (11) fruits; and (12) miscellaneous including condiments, snacks, and beverages. We identified food groups from food photographs and meal names (Supplementary Figure 3). As it was difficult to identify oil, sugar, and salt content from a food photograph directly, we tagged a meal with an unhealthy label, based on whether the meal contained food that was fried or high in sugar or salt content. It was easy to discern fried food. Regarding high-sugar or high-salt food, we tagged them by considering whether or not the meal included a sweetened sugar beverage (identified by the food label in beverage products), sauces, or pickles with high-salt content.

Some meals were also excluded if sufficient information was not available to determine the food group. Finally, 18,435 valid meals from 812 food outlets (Figure 4) were recognized after interpretation, including five types of meals: staple (ST), set meal (SET), noodles and dumplings (ND), western fast-food (WFF), and healthy light recipes (HLRs). Subsequently, we calculated the category of every food outlet according to the rules presented in Appendix 1 in the Supplementary Material, and calculated the TUHII, HII, UHII, and health weight for every food outlet according to the abovementioned equations. For comparison, we standardized the TUHII, HII, and UHII by Z-score.

### Statistical Analysis

For the differences of healthiness and nutrition among the different meals, a descriptive analysis was performed, and the healthy score and DDS were separately described by means and standard deviations. The homoscedasticity of the healthy score and DDS were tested by Levene's test. If the Levene's test indicated homoscedasticity, the difference in healthiness and nutrition among different meals was verified using the analysis of variance (ANOVA), otherwise verified by Welch's ANOVA analysis (66).

**Figure 5:**
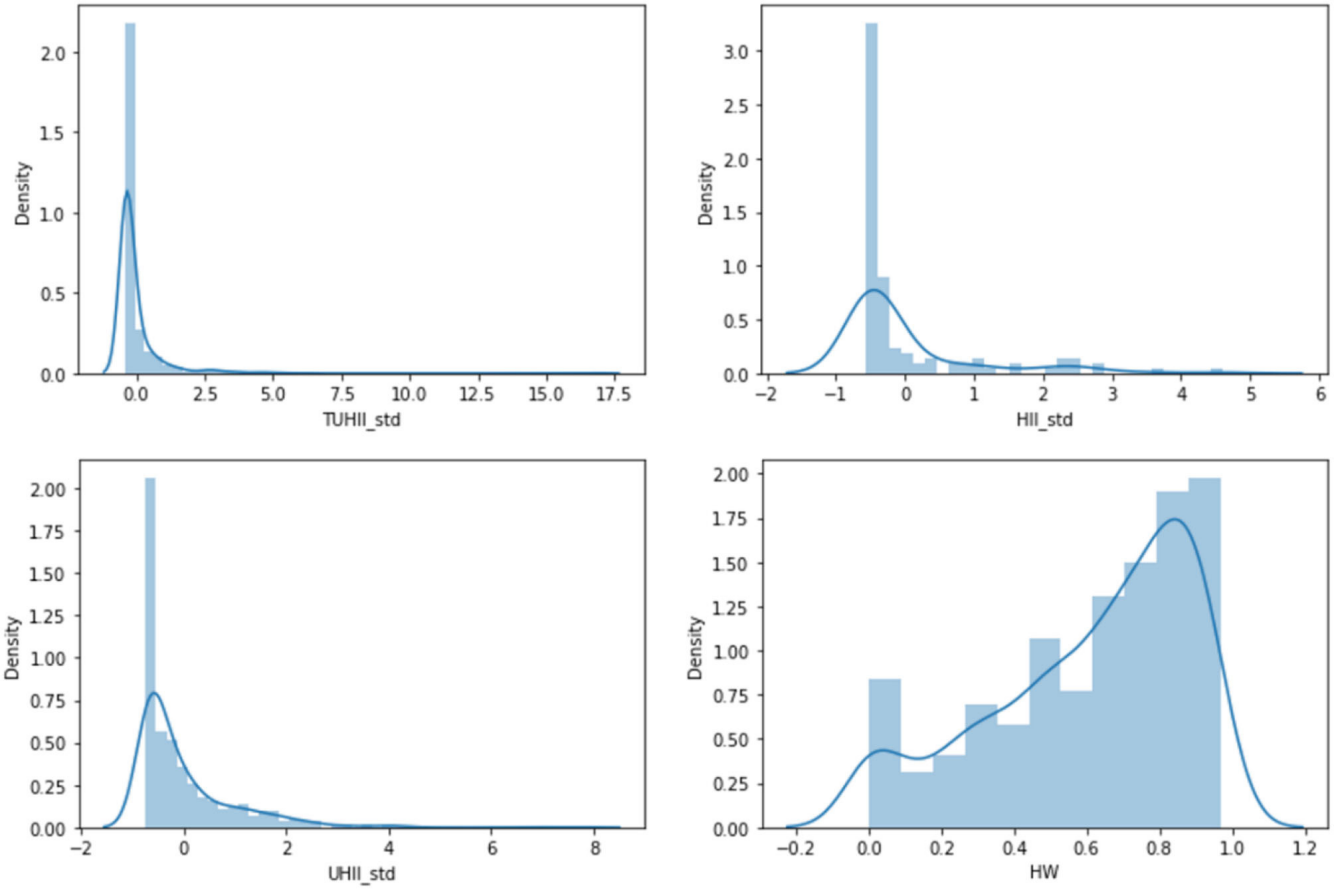
The histogram of standardized TUHII, HII, UHII, and health weight.

To study the differences in unhealthy impact among different food outlets, a descriptive analysis was performed, and the TUHII, HII, UHII, and health weight were separately described by the mean and standard deviation. Considering the distribution of health weight, TUHII, HII, and UHII (Figure 5), we quartered the health weight and labeled them as healthy^3^ (H, W ≥75% quantile), relatively healthy (rH, median ≤ W <75% quantile), relatively unhealthy (ruH, 25% quantile ≤ W < median), and unhealthy (uH, W <25% quantile). We quartered the standardized TUHII, HII, and UHII, and labeled them as follows: Q1 (the standardized TUHII, HII, or UHII <25% quantile), Q2 (25% quantile ≤standardized TUHII, HII, or UHII < median), Q3 (median ≤ the standardized TUHII, HII, or UHII <75% quantile), and Q4 (the standardized TUHII, HII, or UHII≥75% quantile). The difference of standardized TUHII, HII, UHII and health weight among different food outlets was analyzed by the same method as that in meals analysis (66).

The spatial pattern of TUHII, HII, and UHII were analyzed by spatial autocorrelation using the inverse distance to specify the neighborhood relationship and tested by the Global Moran's I. The regional patterns of TUHII, HII, and UHII were verified by kernel density analysis, and the spatial resolution was 30 m. Further, we compared the differences of aggregation results of TUHII, HII, and UHII in different subdistricts. We tested the correlation of the results measured by the counting method for food environment availability and TUHII, HII, and UHII in different subdistricts using Spearman's correlation analysis. Subsequently, we then compared the differences between them. All the statistical analyses were conducted using SPSSAU (Beijing, China), and parts of data were processed in Python 3.6. A *p*-value of < 0.05 was regarded as significant. The final maps were drawn using ArcGIS 10.6.

### Results

#### Differences in Healthiness and Nutrition Among Various Meals

The number distribution of the different meals with different healthy score (*l*) and DDS are listed in Supplementary Table 1. The table shows that two most popular meals were SET and ND types, accounting for nearly 80% of total meals. The DDS of most meals (~69% of total meals) were 4 or 5. More than 60% of meals were healthy (the healthy score equaled 0), about half of which were ND meals. The healthy score of most unhealthy meals (at least one unhealthy food in the meal) was 1, of which SET meals were of majority. In all types of meals, ST and WFF meals had the highest proportion of unhealthy meals.

The result of Levene's test showed that the variance for the healthy score among five types of meals were not equal (F = 711.16, *p* < 0.01). Overall, there were significant differences in the healthy score among all types of meals according to Welch's ANOVA test (Supplementary Table 2). Specifically, the average healthy score (0.45) of WFF meals was the highest (0.79), and that of HLR meals was the lowest (0.14). In the five types of meals, the average healthy score of only two types of meals was below the total average (HLR, 0.14; ND, 0.30). The average healthy scores of ST (0.64) and WFF (0.79) were significantly higher than the total average.

The variance in DDS among five types of meals were not equal too (F = 79.28, *p* < 0.01). There were significant differences in DDS among all types of meals (Supplementary Table 3). Specifically, the average DDS of HLR was the highest (6.34) and that of ST was the lowest (4.58). Among the five types of meals, the average DDS of only ND (4.70) and ST were less than the total average (4.82). Surprisingly, the average DDS of HLR was the highest, followed by WFF.

#### Differences in the Impact Degree Among Various Food Outlets

After aggregation, there were 123 healthy food outlets and 689 food outlets at different unhealthiest levels, including six types of food outlets: the unknown type (UN) and five other categories (same as the meals). In total, regardless of the total food outlet samples or healthy and unhealthy food outlet samples, the number of ND and SET food outlets was the highest (Supplementary Tables 4–6).

Among the total food outlets, there were significant differences in standardized TUHII (Supplementary Table 4). The average standardized TUHII of WFF (0.25), ST (0.22), and SET (0.13) food outlets were significantly higher than the total average standardized TUHII (0.00). Further, the average standardized TUHII of HLR (-0.28), ND (-0.11), and UN (-0.30) food outlets were considerably lower than the total average standardized TUHII. Specifically, the mean standardized TUHII in the Q1 (−0.39), Q2 (−0.37), and Q3 (−0.25) groups were significantly lower than the total average standardized TUHII. Moreover, the mean standardized TUHII of all types of food outlets were nearly equal to that in the corresponding Q1, Q2, and Q3 groups; however, those in the Q4 group were different. The mean standardized TUHII of HLR (0.24) and UN (0.33) food outlets were significantly lower than the total average standardized TUHII (1.02) in the Q4 group. The mean standardized TUHII of ND (0.76) and WFF (0.80) food outlets were considerably lower and that of SET (1.34) food outlets was considerably higher than the total average standardized TUHII in the Q4 group.

Regarding healthy food outlets, Welch's ANOVA test could not be conducted owing to lack of food outlets in some categories. Thus, we simply listed the number of healthy food outlets and the mean and standard deviation of the standardized HII in each quartile group (Supplementary Table 5). There was no Q1, Q2, and Q3 groups in HLR and ST food outlets and no Q1, Q3, and Q4 groups in WFF food outlets. The number of HLR, ST, and WFF of food outlets was less than five. Thus, it was difficult to reasonably assess the position of the mean standardized HII of these food outlets relative to the total average of standardized HII.

Among unhealthy food outlets, there were significant differences in standardized UHII (Supplementary Table 6). In total, the mean standardized UHII of UN (−0.41) and WFF (−0.28) food outlets were significantly lower, and that of ST (0.13) food outlets was significantly higher than the total average standardized UHII (0.00). Specifically, the mean standardized UHII of various food outlets in the Q1 and Q2 groups were similar to the total average standardized UHII in the Q1 and Q2 groups. In the Q3 group, the average standardized UHII of ST (-0.06) and WFF (−0.05) food outlet was considerably higher than the total average of standardized UHII (−0.12). In the Q4 group, the average standardized UHII of ST (1.83) and UN (1.6) food outlets were significantly higher, and those of HLR (0.68) and WFF (0.61) food outlets were significantly lower than the total average standardized UHII (1.38).

There were significant differences of health weight across various types of food outlets (Supplementary Table 7). The mean health weight in HLR (0.85) food outlets was significantly higher and those of WFF (0.42) and ST (0.46) food outlets were significantly lower than the total average health weight (0.61). The average health weight of various food outlets in the H, rH, and ruH groups were approximately equal to the total average of health weight in the corresponding groups, and the difference of health weight in various food outlet in uH group was similar to the differences in total.

#### Characteristics of the Spatial Distribution for TUHII, HII, and UHII

As shown in Supplementary Figure 4, Global Moran's I test (Z = 0.12, *p* = 0.91) for TUHII revealed that there was no significant spatial variance indicating that the TUHII of food outlets were distributed randomly. Moran's index of HII was 0.87 (Z = 6.67, *p* < 0.01). Moran's index of UHII was 0.09 (Z = 2.02, *p* = 0.04).

For local spatial differences, the kernel density analysis result recognized the similar amount and distribution of areas with high TUHII and UHII and only one area with high HII, which also was the same area with TUHII and UHII (Panels B, D, and F in Supplementary Figure 5). However, the different areas with high TUHII, HII, and UHII were influenced by different types of food outlets (Panels A, C, and E in Supplementary Figure 5). For aggregation results of standardized TUHII (Supplementary Figure 6), the different subdistricts were highly influenced by different food outlets. Specifically, the sum of standardized TUHII of HLR food outlets was considerably higher in the ZLL subdistrict than in others. Further, the sum of standardized ND and ST food outlets were relatively higher in the XJK subdistrict than in others. Moreover, the sum of standardized SET food outlets was higher in the YDL subdistrict compared with the others, and the sum of standardized UN food outlets was higher in the ZZY subdistrict than in others. Additionally, the sum of standardized WFF food outlets was comparably higher in the BXG subdistrict than in others. For aggregation results of standardized HII (Supplementary Figure 7), the different subdistricts were also influenced by different food outlets, except the ST and WFF, which were unhealthier than other types of food outlets in the differences analysis of indicators among various food outlets. In the remaining food outlets, the sum of standardized HII of HLR food outlets was considerably higher in the GJK subdistrict than in other subdistricts. Further, the sum of standardized ND food outlets was relatively higher at BXG subdistrict than in others. Moreover, the sum of standardized SET food outlets was pretty higher in the BLZ and BXG subdistrict compared with others. Additionally, the sum of standardized UN food outlets was higher in the BXG and XJK subdistricts than in others. Similar to TUHII and HII, for aggregation results of standardized UHII (Supplementary Figure 8), the different subdistricts were also influenced by different food outlets. However, the number of subdistricts influenced highly was more than that in the case of TUHII and HII. In HLR food outlets, ZZY, BXG, and ZLL subdistricts were highly influenced; in ND food outlets, SG and BXG subdistricts were influenced higher than in other subdistricts. Further, in SET food outlets, XJK and YDL subdistricts were influenced higher than in others, and in ST food outlets, only BLZ subdistrict was highly influenced. Moreover, in UN food outlets, only GJK subdistrict was influenced higher than in others; in WFF food outlets, SG, BLZ, and YT subdistricts were highly influenced.

#### Differences of the Food Environment Measured by the Counting Method and TUHII, HII, and UHII in Different Sub-districts

The results of Spearman's correlation test (Table 3) showed that there were no significant correlations between the results of food environment measured by the counting method and TUHII, HII, and UHII in different sub-districts. This means that the availability measured by counting at different subdistricts were completely different from that measured by corresponding new indicators.

**Table 3:** Spearman's correlation test between results of food environment measured by the counting method and TUHII, HII, and UHII in different subdistricts.

**Samples**	**Coefficient**	***p***
Total food outlets	0.134	0.713
Healthy food outlets	−0.061	0.868
Unhealthy food outlets	0.158	0.663

**Figure 6:**
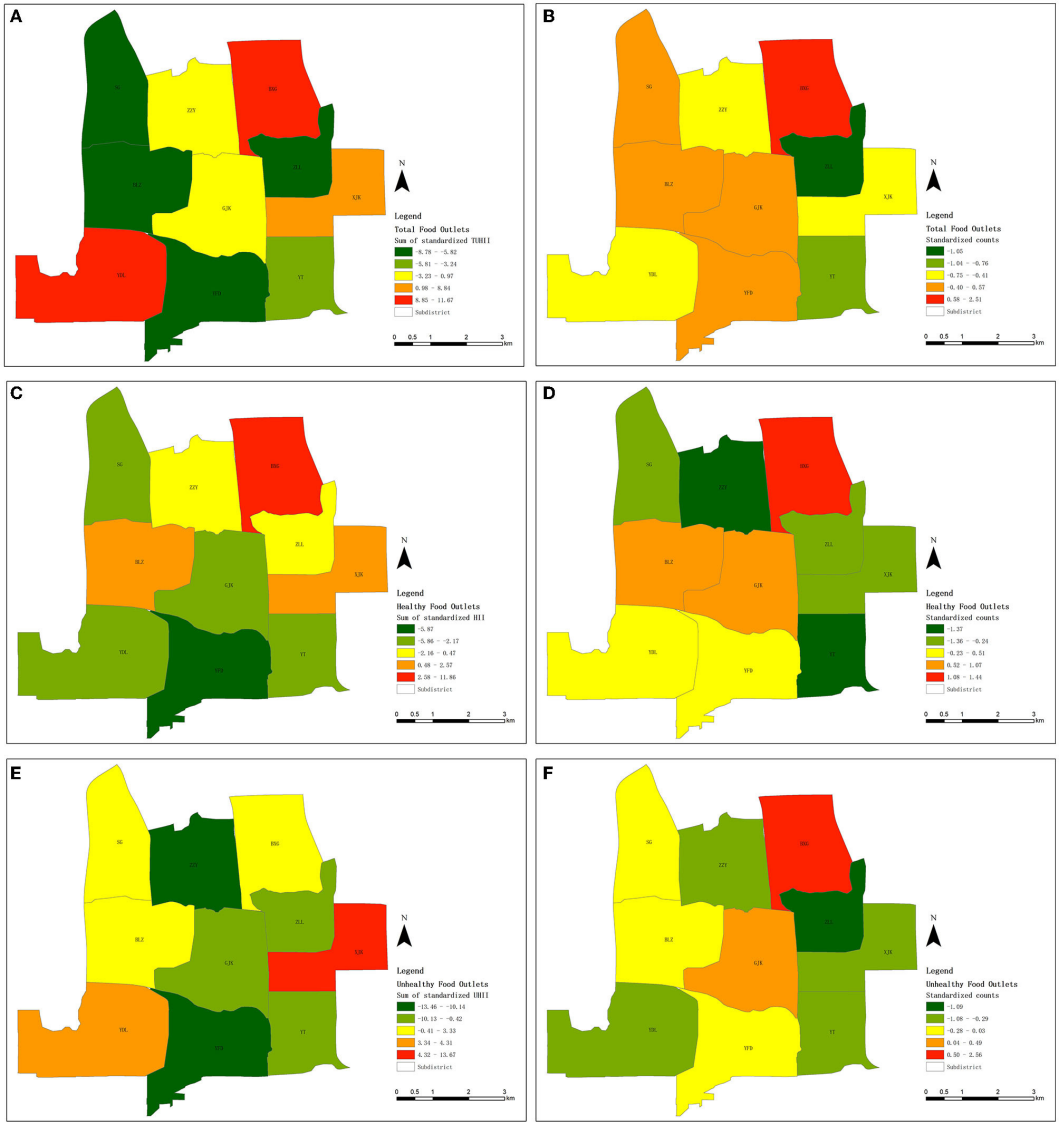
Comparison between two measurements: **(A, C, E)** new method, and **(B, D, F)** old method (measuring the food environment by counting food outlets in an area). ND, Noodles and dumplings; WFF, Western fast-food; SET, Set meal; ST, Staple; HLR, Healthy and light recipes; UN, Unknown. BLZ, Balizhuang; BXG, Beixiaguan; GJK, Ganjiakou; SG, Shuguang; XJK, Xinjiekou; YFD, Yangfangdian; YDL, Yongdinglu; YT, Yuetan; ZLL, Zhanlanlu; ZZY, Zizhuyuan. **(A)** The standardized TUHII summed by subdistricts. **(B)** The standardized counts of total food outlets summed by subdistricts. **(C)** The standardized HII summed by subdistricts. **(D)** The standardized counts of healthy food outlets summed by subdistricts. **(E)** The standardized UHII summed by subdistricts. **(F)** The standardized counts of unhealthy food outlets summed by subdistricts.

Specifically, the consistent measurement results for total food outlets (Panels A and B in Figure 6) were in the BXG, ZZY, ZLL, and YT sub-districts. Further, consistent results for healthy food outlets (Panels C and D in Figure 6) were in the BXG, SG, and BLZ sub-districts, and those for unhealthy food outlets (Panels E and F in Figure 6) were in the SG, BLZ, and YT sub-districts. Compared to the result measured by standardized TUHII, the results measured by the counting method in the SG, BLZ, and YFD sub-districts were considerably overestimated, and the high risk in the YDL sub-district was not recognized in the result of counting method. Moreover, compared to the result measured by standardized HII, the results measured by the counting method in the GJK sub-district was overestimated, and the results in the ZZY and XJK sub-districts were underestimated. For unhealthy food outlets, the high-risk areas recognized by two methods were different (observe the red color area between E and F in Figure 6): the high risk recognized by standardized UHII was in the XJK subdistrict, but the high risk recognized by the counting method was in the BXG subdistrict. Additionally, compared to the result measured by standardized UHII, those measured by counting in the GJK and YFD subdistricts were overestimated, and the result in the YDL subdistrict was underestimated.

## Discussion

### Findings

To the best of our knowledge, this is the first study to examine the OPFE in China. We proposed an analytical framework in which food is placed in the center linking the food environment and consumers as one of the key elements of the food environment. We first explored the differences of the healthiness and nutrition in different types of food because takeaway food has been typically defined as unhealthy by default as a type of fast-food. From a healthiness perspective, most takeaway foods for a single meal were healthy in 10 subdistricts. The highest ratio of unhealthy food in a single meal was in the ST type of meals, which is reasonable because citizens of Beijing are used to having at least one kind of fried food in ST meals at breakfast, such as fried bread sticks or rings. Although the WFF meal had a higher ratio of unhealthy food in a single meal than other meals, the amount of healthy food in WFF meals was a little more than that of unhealthy food, which was unexpected. Further, surprisingly, there was unhealthy food in HLR meals, which normally featured healthy foods. From the perspective of nutrition, we tested that there was a significant difference among different types of meals, but the remaining meals, except HLR, did not differ significantly in the mean DDS. Notably, the DDS of more than 90% meals were >4. For the population (household DDS, women DDS, or children DDS), a DDS value <4 would be considered low dietary diversity (67). Thus, the DDS of most takeaway food on a single meal in our study area met the daily standard of population. Considering the distinctive characteristics of WFF and HLR meals, the parameters we selected could depict the features of meals correctly. Thus, the above results answered the first research question, and provided new evidence that takeaway foods cannot be identified unhealthy directly in China.

At the food outlet level, there were only 123 healthy food outlets, accounting for <15% of the total food outlets. Even among the HLR food outlets—which were the representative of healthy food outlets—the healthy food outlets were only one-fifth of the total HLR food outlets. This result was expected to be similar with the initial impression that most fast-food is unhealthy. Moreover, the healthy food outlets may have only been healthy at the time of data collection, and this also might be related to the method of healthiness we designed. We measured the healthiness of food by proxies, which were usually add-on sale. Thus, a reasonable explanation would be that these healthy food outlets did not sell unhealthy food at the time of data collection. Similarly, even among unhealthy food outlets, only a few sold healthy food items (Supplementary Table 7). Furthermore, the unhealthy impact degree of various food outlets was different. Interestingly, the mean standardized TUHII of WFF food outlets was higher than the total average standardized TUHII. However, the mean standardized HII and UHII of WFF food outlets were lower than the corresponding total averages of standardized HII and UHII. This result indicated the following: (1) the actual unhealthy impact of WFF food outlets was lower than the expected, regardless of the healthy or unhealthy groups samples; and (2) the number of unhealthy foods in WFF meals was more than in any other type of meals (Supplementary Table 1), especially in terms of WFF meals with two unhealthy foods.

According to the results of kernel density analysis and aggregation by subdistricts, various high-risk areas were identified by different food outlet samples. Notably, the average number of high-risk areas identified by total food outlets was lower than that identified by healthy and unhealthy food outlets. This indicates that the actual number of high-risk areas might be underestimated in the mixed food outlet samples. The comparison between the aggregation results measured by the counting method and the method proposed in this study showed that the unhealthy impact of food outlets was incorrectly estimated by the counting method in approximately two-thirds of the subdistricts.

All analyses in the above two paragraphs could answer the second research question and the proposed indicators could be potentially useful tools for monitoring the food environment. Moreover, many researchers have explored the reasons behind the inconsistent conclusion: the data sources (68–74); the measurement or selection of the food environment (22, 69, 75, 76); neighborhood effect (77); study design and quality (28, 78); the change of the food environment (79); the temporal and spatial uncertainty (80); and the complexity (32). However, few studies have deconstructed the inner characteristics of the food environment. The current results shed light on these inconsistent conclusions.

### Limitations

This study had several limitations. First, we cannot assess the performance of the indicator on the association between the food environment and human health, owing to the lack of population data. Although we compared the differences between the two results of the food environment measured by the counting method and the indicators proposed in this study, we cannot conclude that our methods were better than the counting method in assessing the association between the food environment and human health. Thus, population health data should be collected in the future to verify the indicator performance.

Second, the indicator was designed based on single-meal foods, which means that we cannot identify the food attributes of F_BBQ and POT food and corresponding food outlets because they were self-selected by consumers, seafood, and corresponding food outlets because they were sold by weight. Moreover, the characteristics of snacks and beverages were not identified owing to a lack of data. While calculating the healthy score, using proxies would inevitably involve uncertainty. Thus, in future research, the indicator should be revised, or a new indicator should be developed for F_BBQ, POT, and SF types of food and food outlets. The indicators proposed in this study should also be applied to snacks and beverages in the future. FDPs and the government should cooperate to solve the uncertainty problem caused by proxies.

Third, regarding the classification system of food outlets and meals, although we tried to cover all types of food, we cannot be sure that the classification system can be generalized because this system was developed based on the data from a small area in Beijing. More work is needed to expand its boundary of application to the whole food environment, comprising all varieties of food outlets and providing food service. Additionally, the cutoff of the ratio of the maximum and minimum(MMR, a parameter to identify the category of the food outlet; Appendix 1 in the Supplementary Material) in rules for calculating the type of food outlets from that of food was set referring to authors' data observations. A theoretical method should be developed to calculate the type of food outlet from that of food in the future.

Fourth, the DDS is designed to assess a personal or a family's access to nutrition, such as nutrient adequacy and overall diet quality, over the preceding days or during a week. This is possibly the first time DDS is used to estimate the food diversity of takeaway food as a part of an indicator for measuring the food environment. Nevertheless, we cannot verify the relationships between DDS in a day and that in a single meal. Hence, more research is needed to verify the association of the single-meal DDS and individual DDS.

Finally, the method of feature extraction by artificial interpretation is inefficient. As this is the first study to examine the OPFE-TF in China and the complexity of Chinese food environment have been tested in other provinces (32), the artificial feature extraction is a good way to guarantee the accuracy of the analysis data. Artificial interpretation can be used at the beginning of the research to help collect data characteristics. Automatic or semi-automated feature extraction, however, should be developed in the future to cater to big data.

## Conclusion

The severity of obesity in China is undisputed (81). While it is not easy to intervene within the context of a population, finding a solution from the food environment perspective is worth the effort. This trial study explored a new methodological framework for the OPFE in China by developing an analytical framework and a measurement indicator to define the healthiness and the health impact weight of food outlets. We built a new food environment research framework based on the evidence from the latest disease burden research, combining the characteristics of China's current food environment, from the perspective of environmental science, and referring to the standard process of EHIs. We redefined the food environment and proposed that food and its physical space are two core elements of the food environment. According to this definition, we extracted four domains of characteristics to describe the basic components of the food environment by referring to the existing methods of dietary quality evaluation in nutrition. Based on the single meal, a common form between human eating habit and selling food on the FDP, we designed an approach including three indicators: TUHII, HII, and UHII. As we stated in the Introduction, the indicators developed in this study were mainly used to monitor the characteristics of OPFE-TF, recognize the high unhealthy food risk areas, and prepare to model the associations between OPFE-TF and human health.

The conclusion included: first, the takeaway foods cannot be identified unhealthy directly in China; second, the indicator constructed based on the analytical framework proposed in this study can depict the food environment better compared with the traditional counting method and identify the high risk of OPFE-TF. The food environment characteristics measured by the new approach proposed in this study is closer to reality. This simple but more meaningful approach makes it useful for large-scale and long-term food environmental change monitoring. More importantly, measuring the nutritional value of food available at food outlets to draw public health implications from an analysis of the food environment (12) would not be beyond the scope of food environment research according to the new food environment definition.

Our work in this area is in its initial phase, and more research is needed to verify the effectiveness of the measurement indicator in assessing the impact of OPFE-TF on human health, with interdisciplinary support from nutrition, geography, environmental science, marketing management, and big data companies. Furthermore, more work is needed in the future to revise the measurement to better monitor the change of OPFE and explore the associations between OPFE-TF and population health in a broad spatial range and a longitudinal cohort to develop healthy cities in China (82).

## Data Availability Statement

The original contributions presented in the study are included in the article/Supplementary Material, further inquiries can be directed to the corresponding author/s.

## Author Contributions

NC conducted the whole analyses and drafted the manuscript. AZ helped with the indicator design, M-PK helped with the spatial analysis, and JY helped with the framework. AZ, M-PK, and JY revised the manuscript. PG helped with the framework, supervised, funded the project, and revised the manuscript. All authors read and approved the final manuscript.

## Funding

This research was supported by two grants from the National Natural Science Foundation of China (Nos. 42090015 and 42071400) and donations from the Cyrus Tang Foundation.

## Conflict of Interest

The authors declare that the research was conducted in the absence of any commercial or financial relationships that could be construed as a potential conflict of interest.

## Publisher's Note

All claims expressed in this article are solely those of the authors and do not necessarily represent those of their affiliated organizations, or those of the publisher, the editors and the reviewers. Any product that may be evaluated in this article, or claim that may be made by its manufacturer, is not guaranteed or endorsed by the publisher.

## Footnotes

^1^ https://www.naics.com/naics-code-description/?code=72251

^2^Please see the introduction of RESSET database at http://www.resset.cn/endatabases.

^3^The “healthy” and other labels here were only used to describe the healthiness of unhealthy food outlets.
