# Supplementary material for: An Indicator Measuring the Influence of the Online Public Food Environment: An Analytical Framework and Case Study
Source: Front Nutr. 2022 Jun 30;9:818374. doi: 10.3389/fnut.2022.818374 (PMC9281549; doi:10.3389/fnut.2022.818374)
Supplement: Supplementary file 1 [file Data_Sheet_1.docx]

Supplementary Material

# Supplementary Figures and Tables

## Supplementary Figures


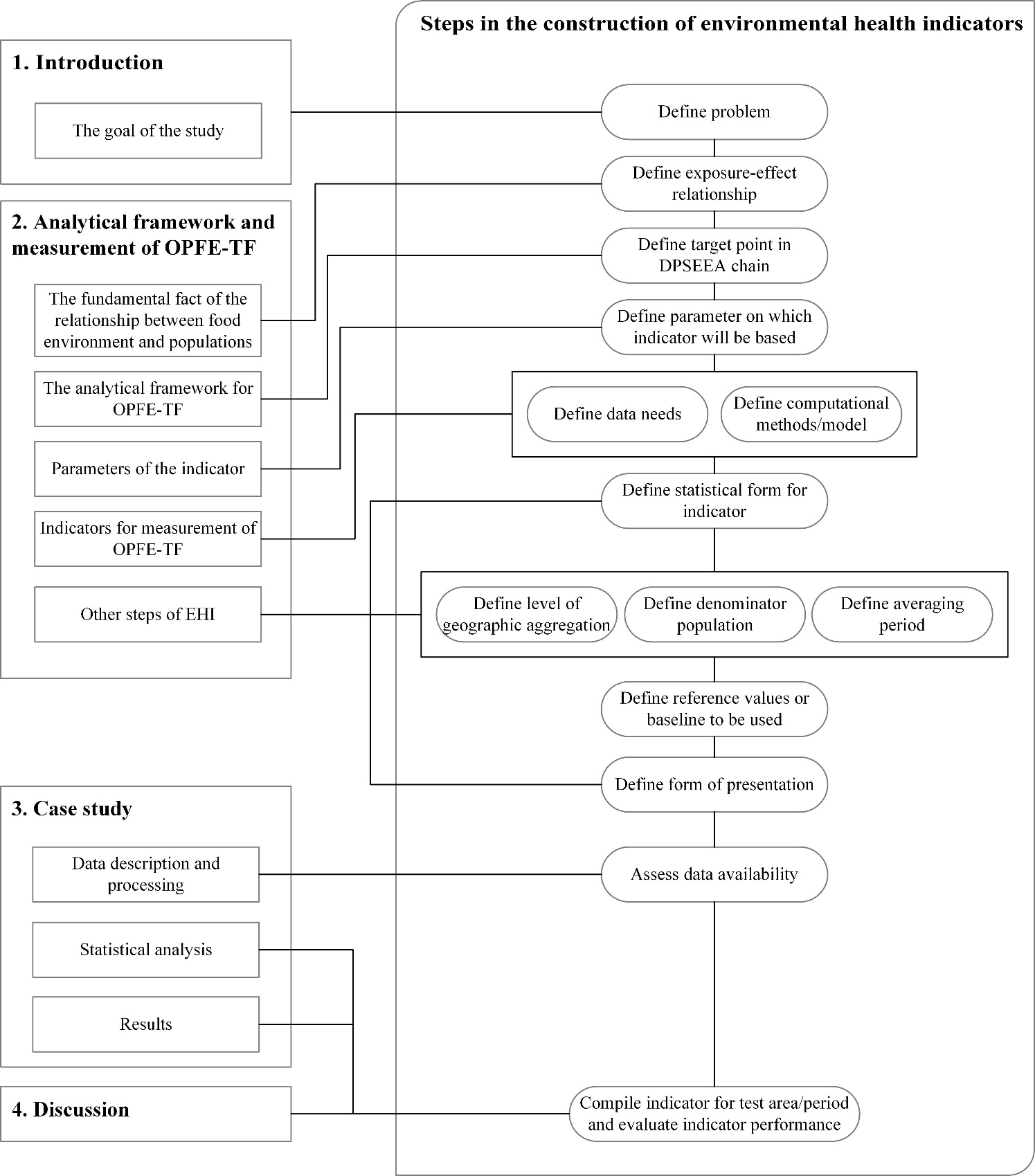


**Supplementary Figure 1. The corresponding relationship between the structure of the article and the construction steps of EHI.** Note: Part of the step (Define data needs and define computational methods/model) was reordered. (Briggs et al., 1996)


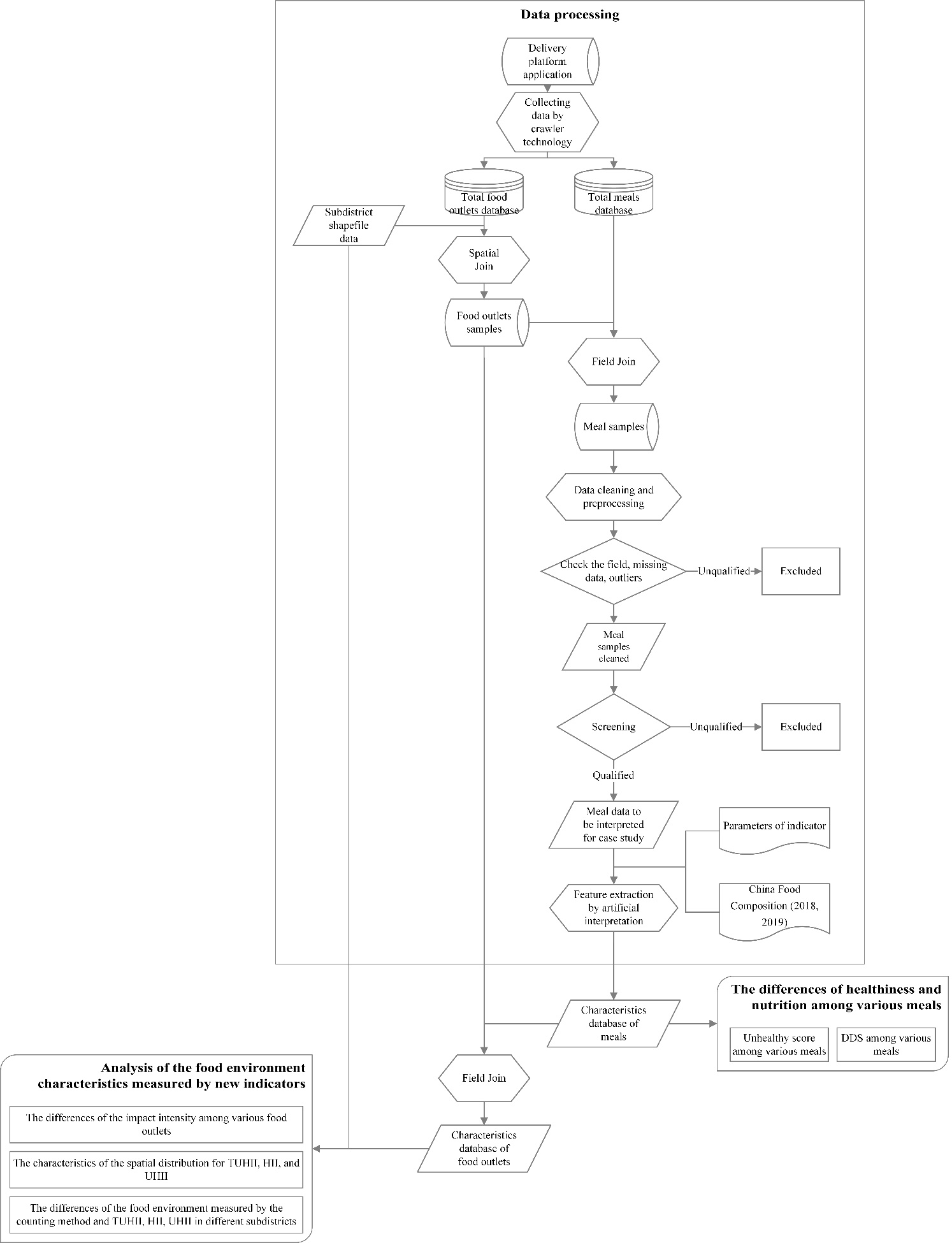


**Supplementary Figure 2. Steps of data processing**


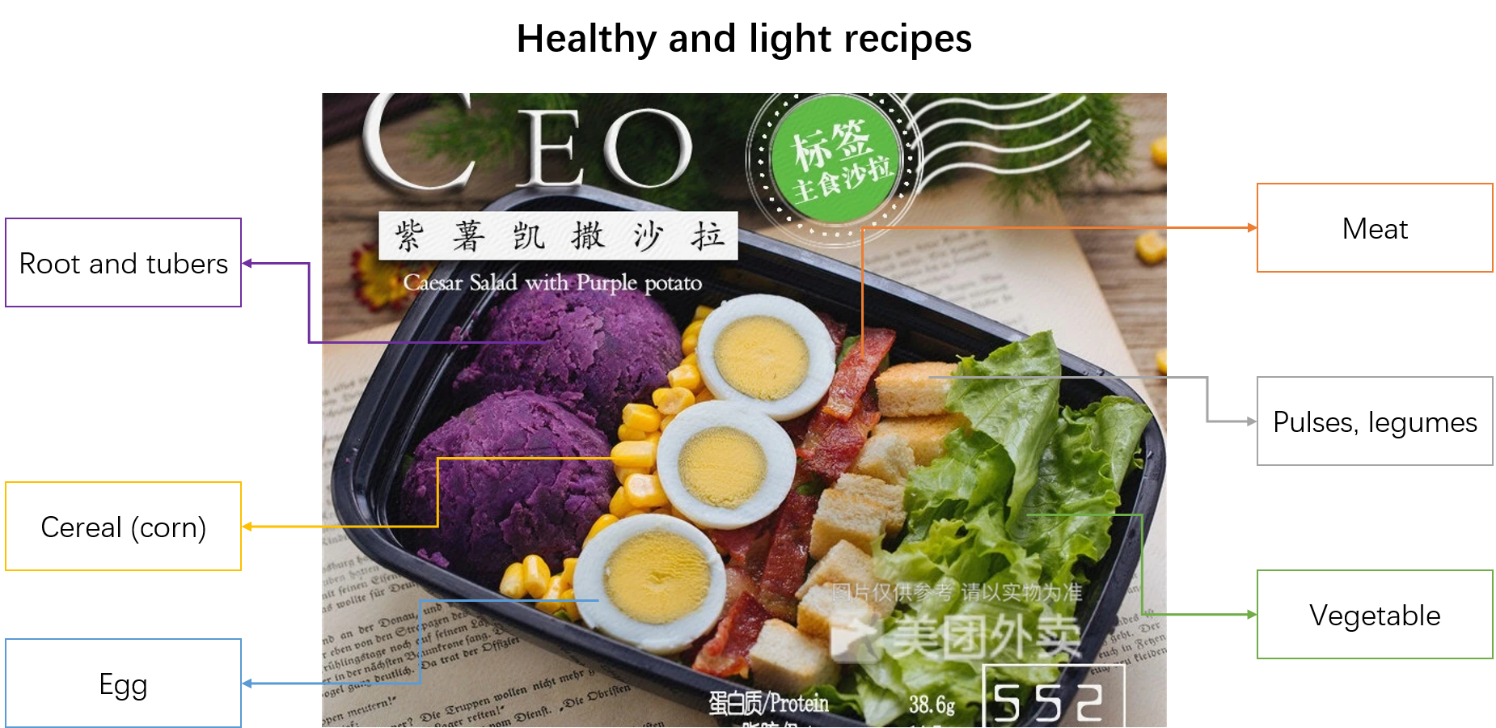


a. Food groups in the healthy and light recipes meals


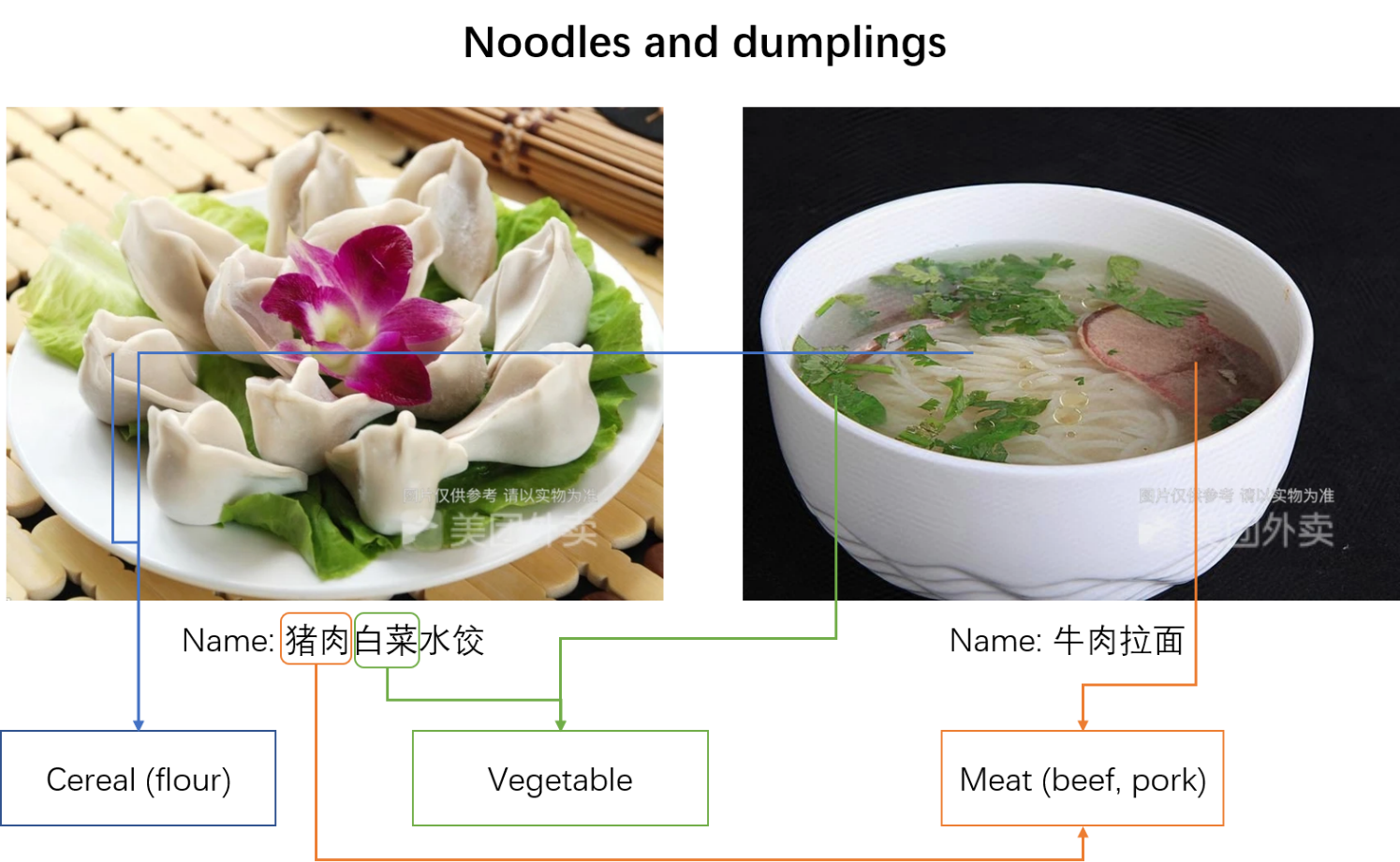


b. Food groups in a noodles and dumplings meal


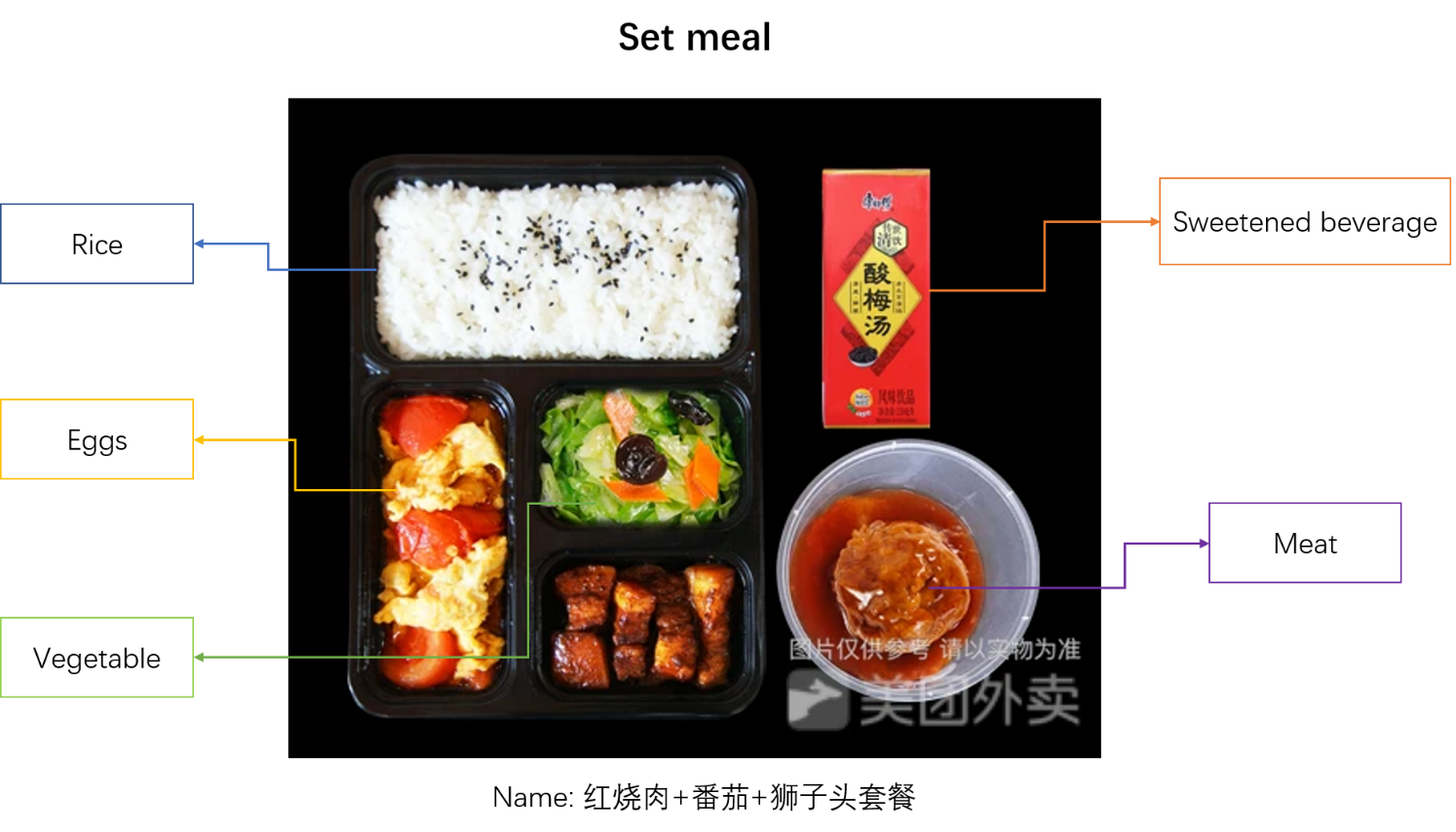


c. Food groups in the set meal photo


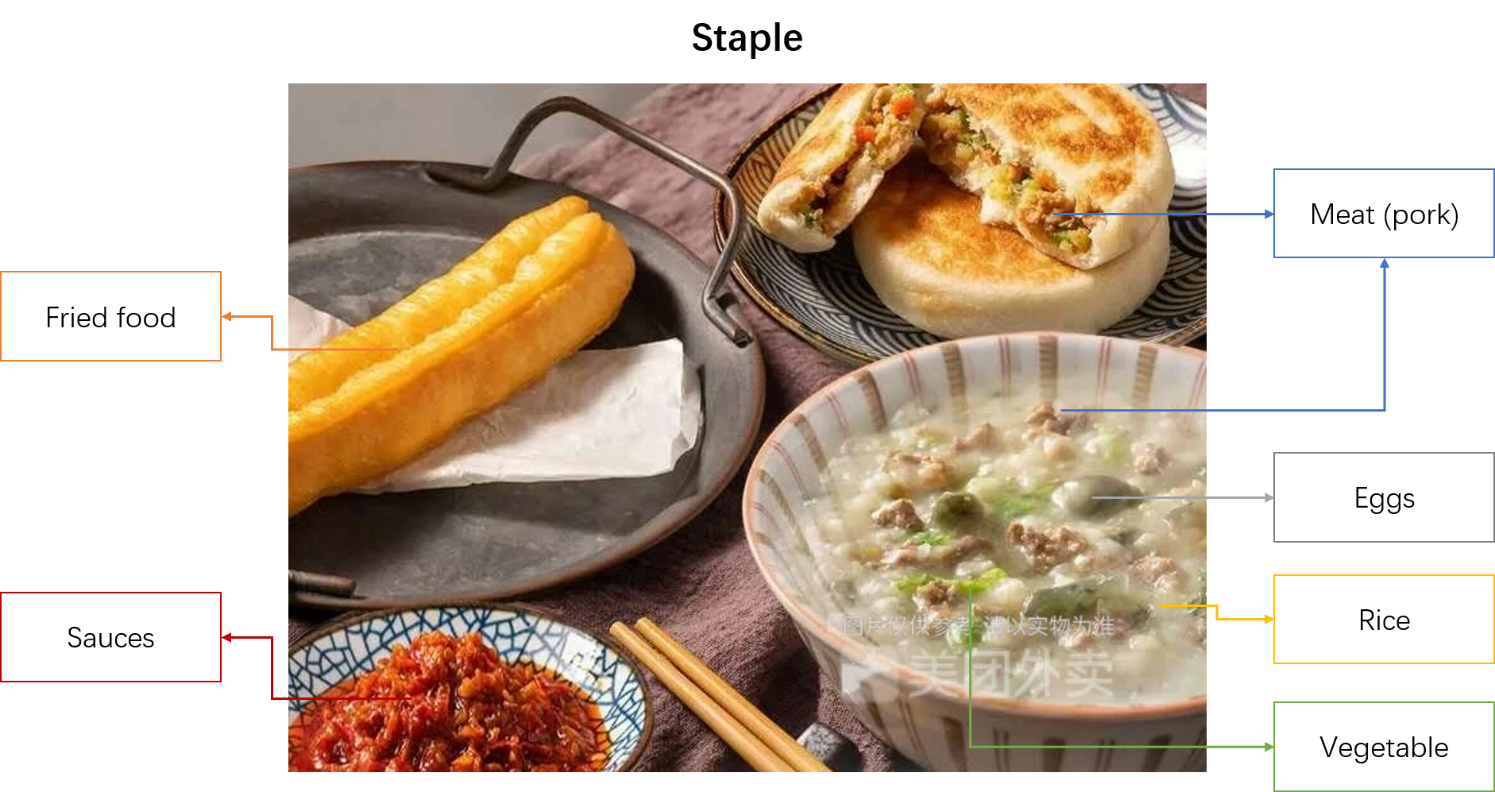


d. Food groups in a staple meal


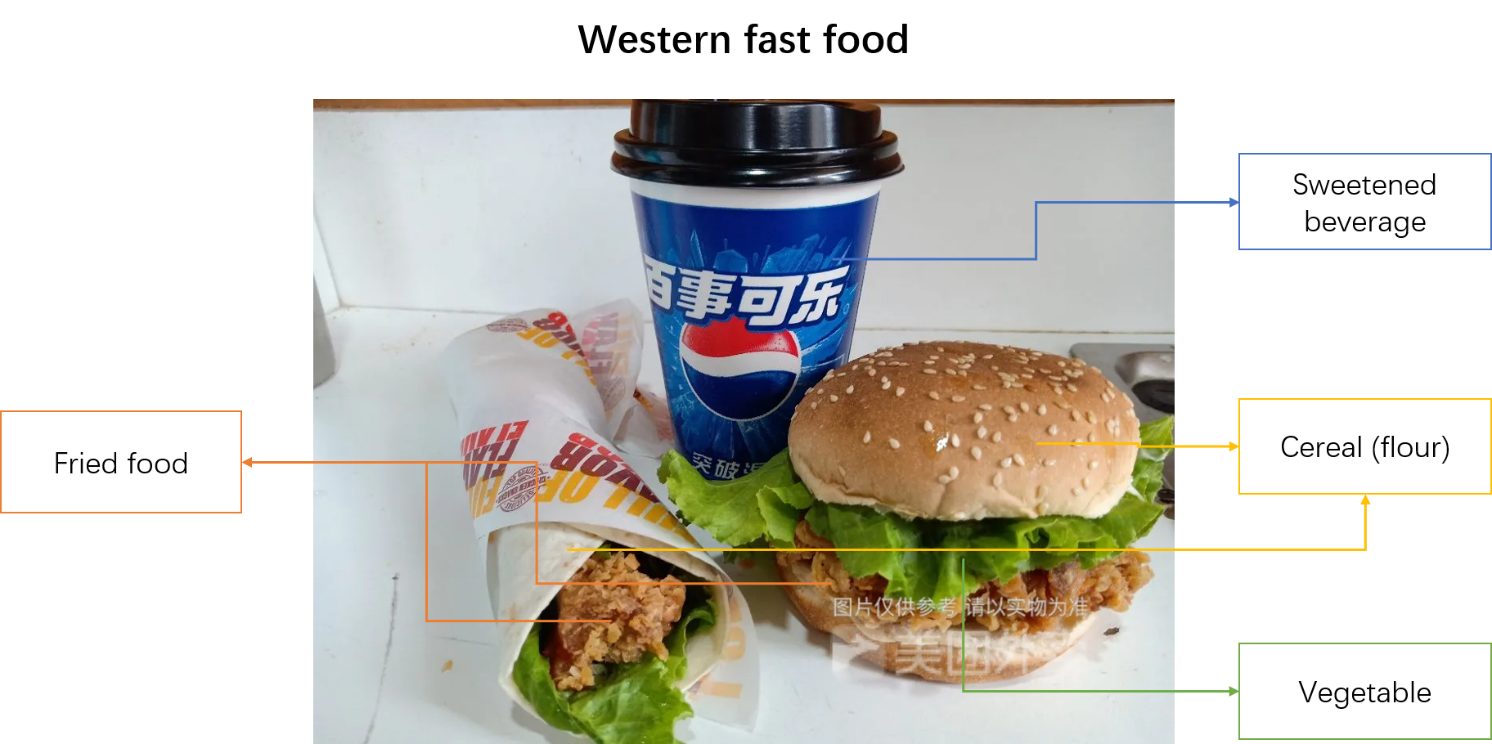


e. Food groups in a western fast food meal

**Supplementary Figure 3. Examples of five categories of meals to be interpreted.** If the photo showed sufficient information, the food group of the meal would be extracted from the photo; otherwise, we would interpret the meal by the photo and the meal name as Figure b shows.

**
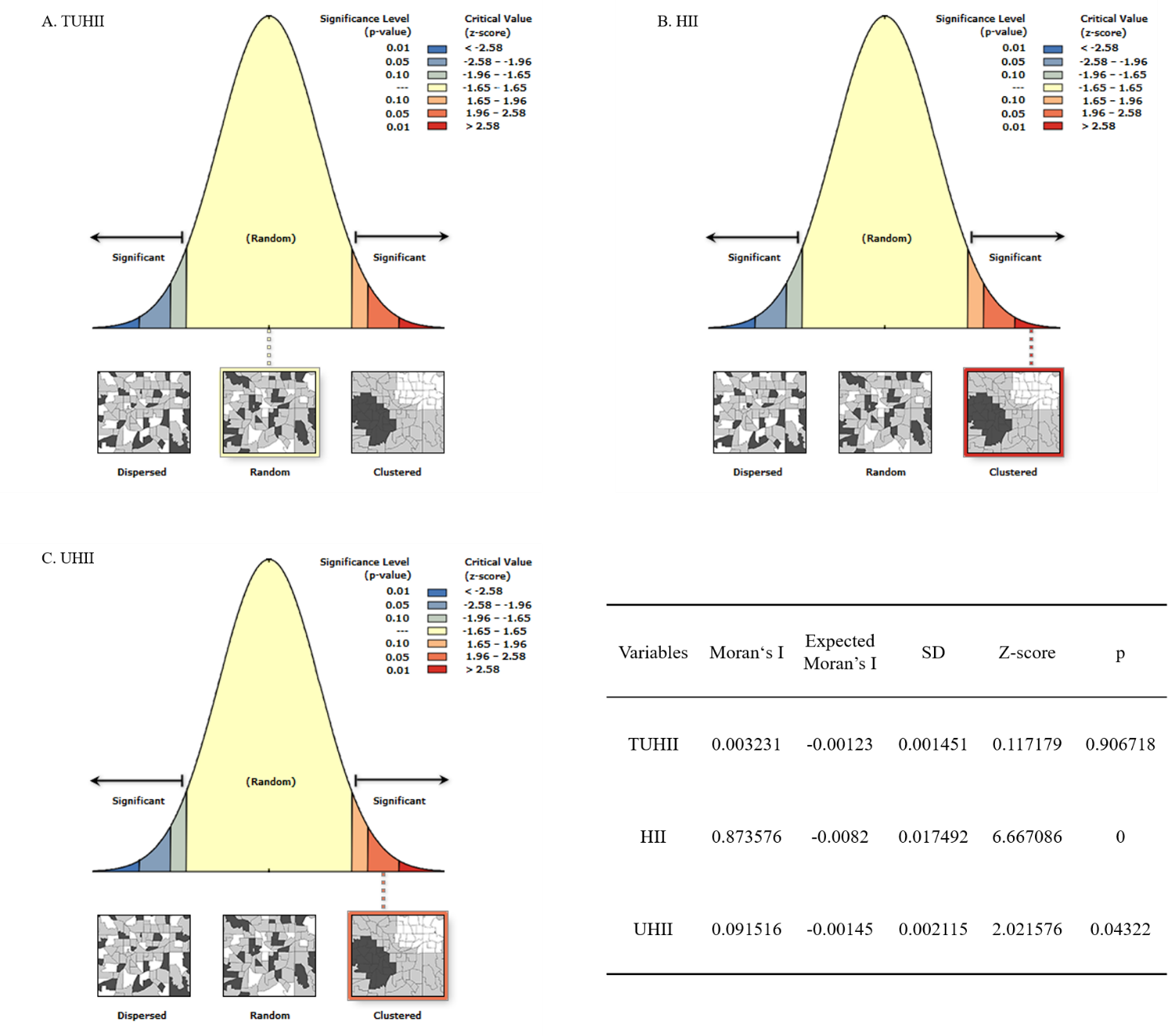
**

**Supplementary Figure 4. Global Moran’s I test results of TUHII, HII, and UHII.**


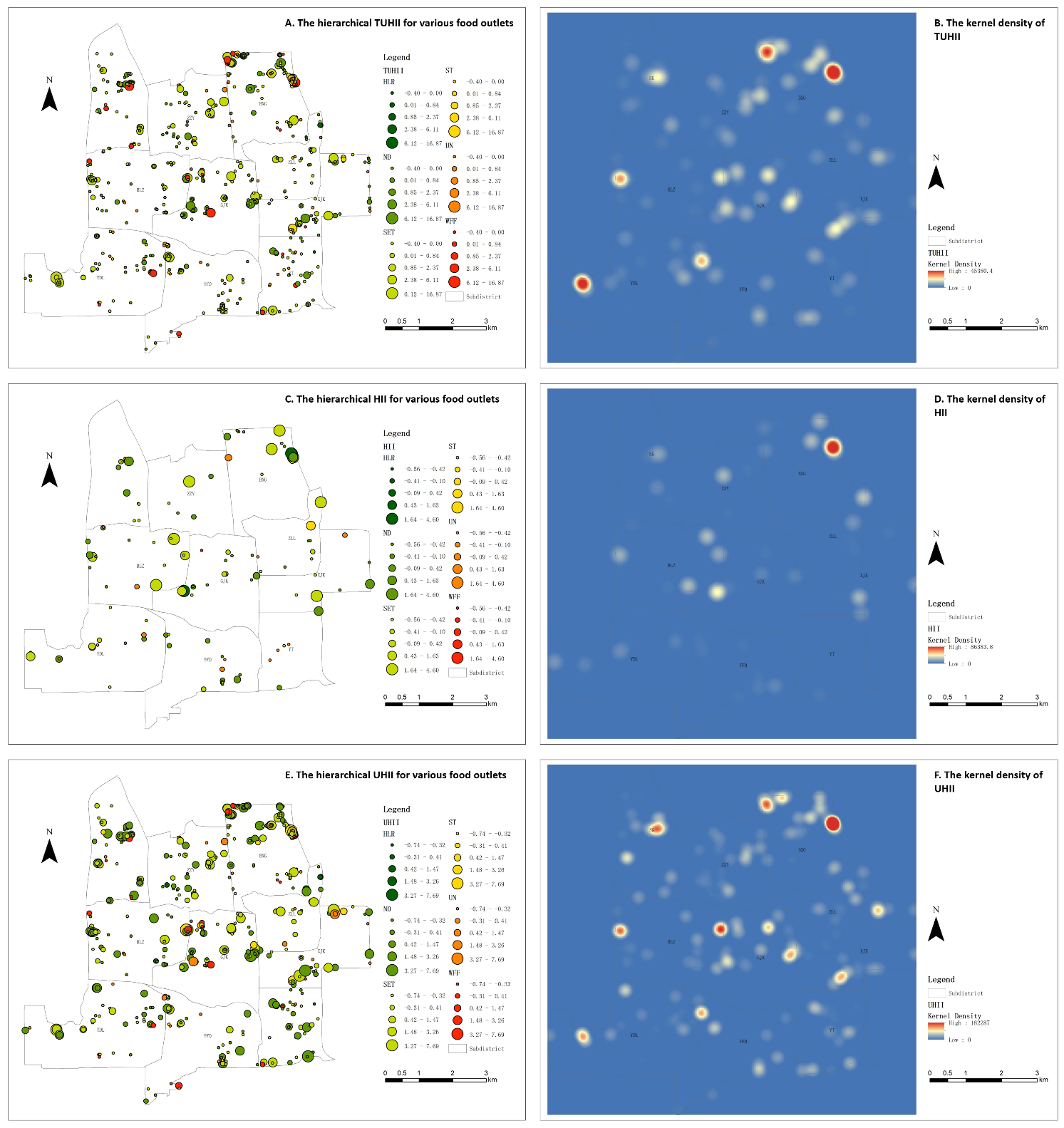


**Supplementary Figure 5. The hierarchical TUHII, HII, and UHII of various food outlets and the kernel density map of TUHII, HII, and UHII.**

ND: Noodles and dumplings; WFF: Western fast food; SET: Set meal; ST: Staple; HLR: Healthy and light recipes; UN: Unknown. BLZ: Balizhuang; BXG: Beixiaguan; GJK: Ganjiakou; SG: Shuguang; XJK: Xinjiekou; YFD: Yangfangdian; YDL: Yongdinglu; YT: Yuetan; ZLL: Zhanlanlu; ZZY: Zizhuyuan.


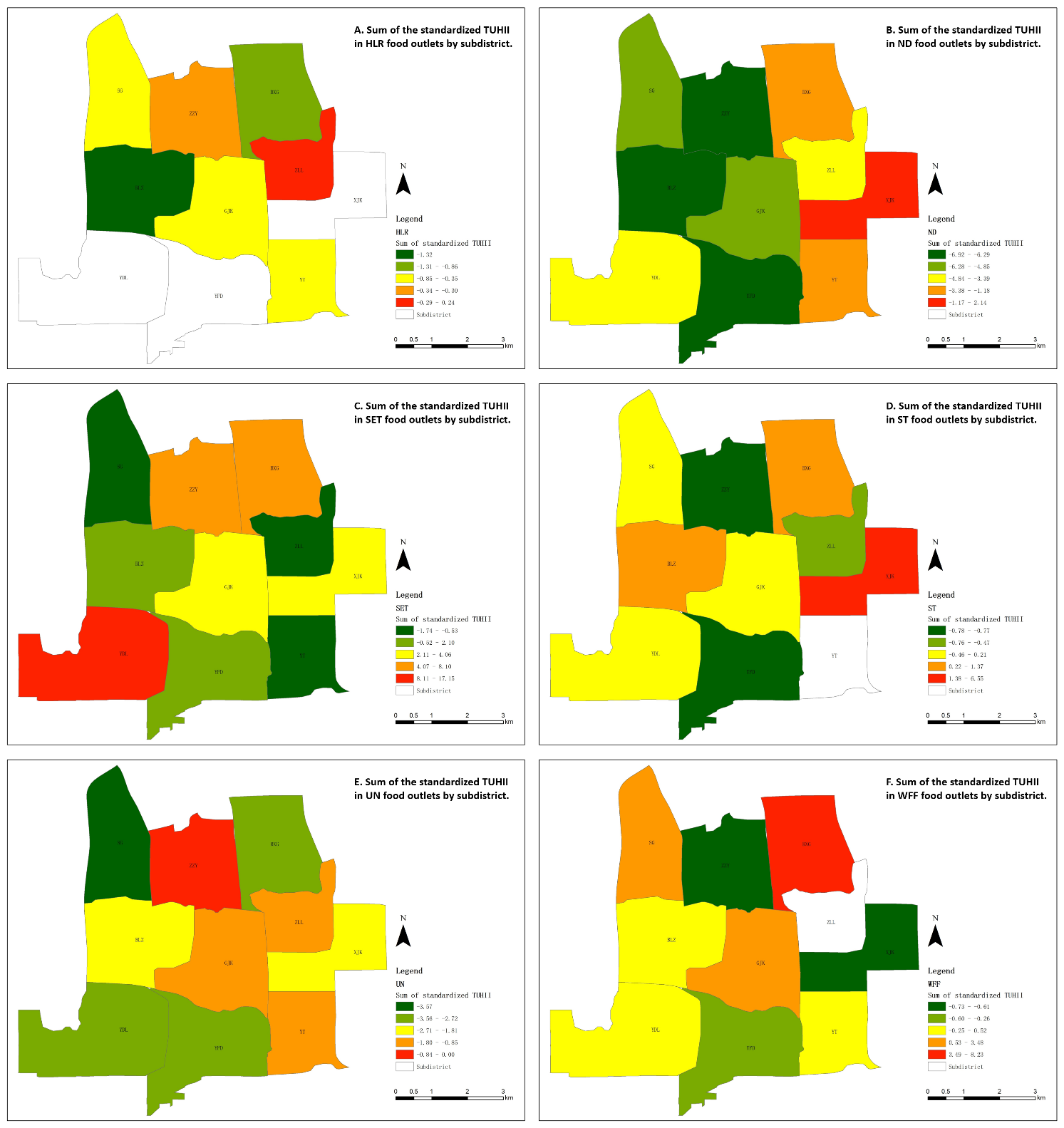


**Supplementary Figure 6.** **The spatial differences of the food environment measured by the sum of the standardized TUHII of every type of food outlet.**

ND: Noodles and dumplings; WFF: Western fast food; SET: Set meal; ST: Staple; HLR: Healthy and light recipes; UN: Unknown. BLZ: Balizhuang; BXG: Beixiaguan; GJK: Ganjiakou; SG: Shuguang; XJK: Xinjiekou; YFD: Yangfangdian; YDL: Yongdinglu; YT: Yuetan; ZLL: Zhanlanlu; ZZY: Zizhuyuan.


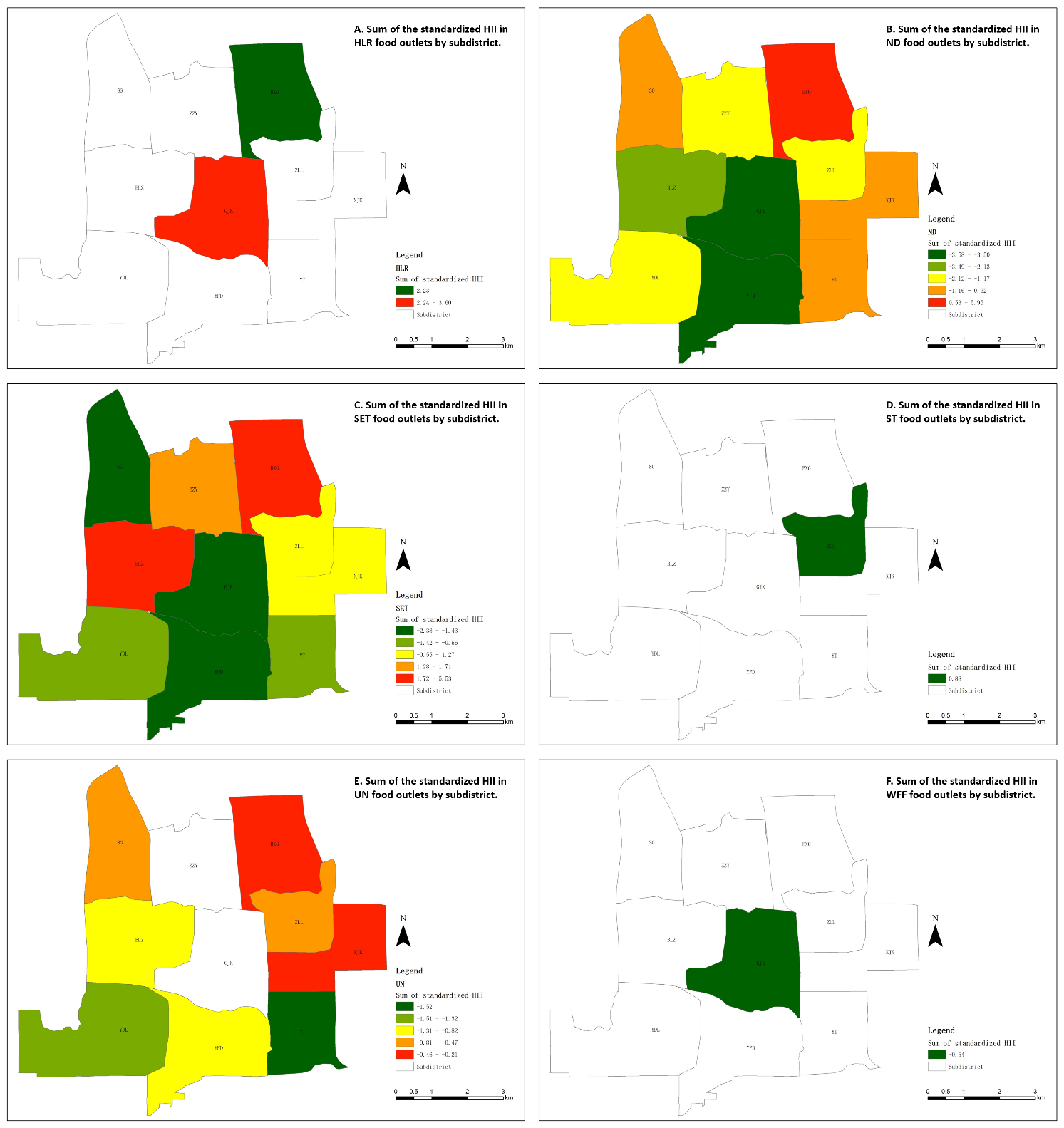


**Supplementary Figure 7.** **The spatial differences of the food environment measured by the sum of the standardized HII of every type of food outlet.**

ND: Noodles and dumplings; WFF: Western fast food; SET: Set meal; ST: Staple; HLR: Healthy and light recipes; UN: Unknown. BLZ: Balizhuang; BXG: Beixiaguan; GJK: Ganjiakou; SG: Shuguang; XJK: Xinjiekou; YFD: Yangfangdian; YDL: Yongdinglu; YT: Yuetan; ZLL: Zhanlanlu; ZZY: Zizhuyuan.


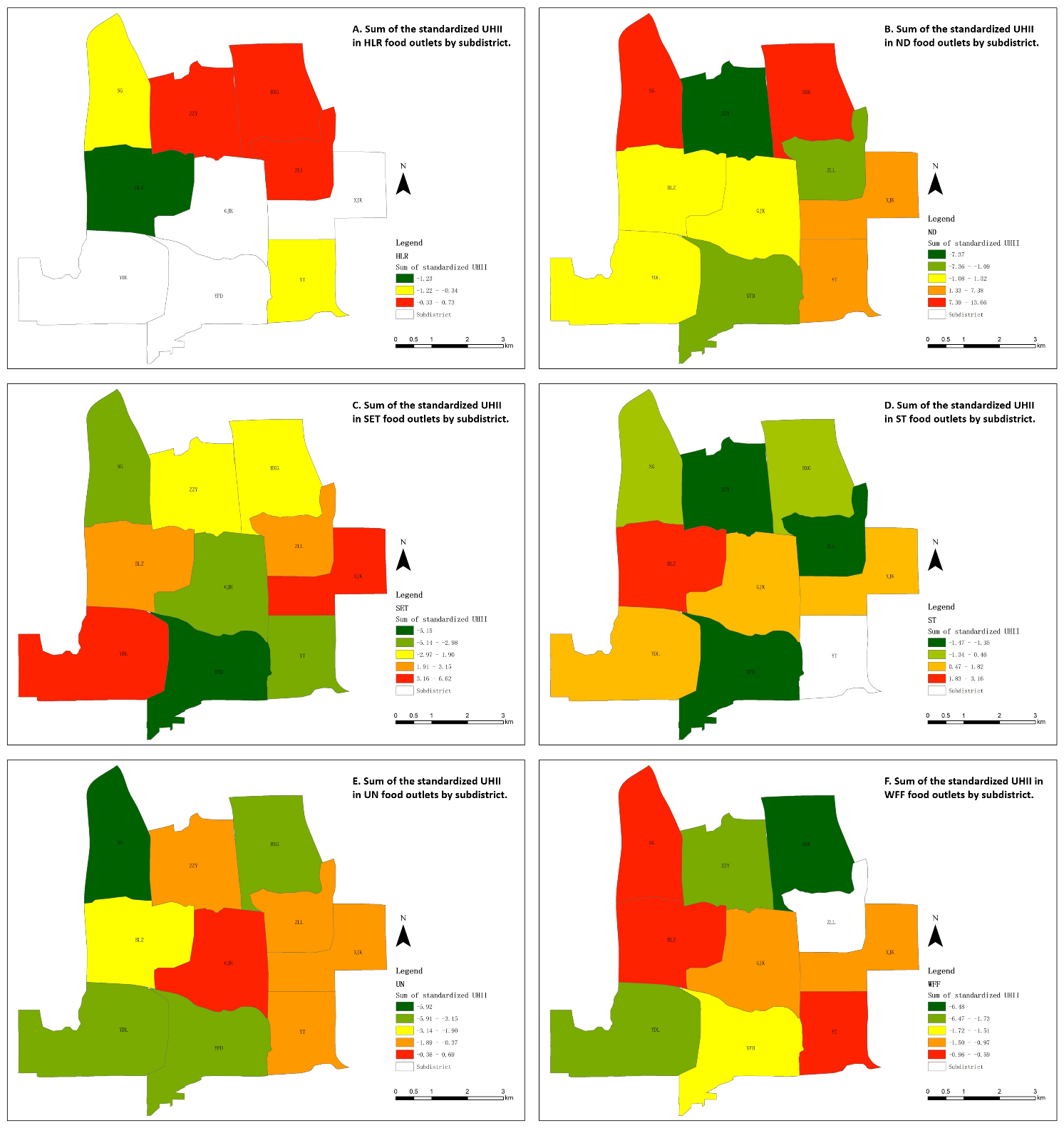


**Supplementary Figure 8.** **The spatial differences of the food environment measured by the sum of the standardized UHII of every type of food outlet.**

ND: Noodles and dumplings; WFF: Western fast food; SET: Set meal; ST: Staple; HLR: Healthy and light recipes; UN: Unknown. BLZ: Balizhuang; BXG: Beixiaguan; GJK: Ganjiakou; SG: Shuguang; XJK: Xinjiekou; YFD: Yangfangdian; YDL: Yongdinglu; YT: Yuetan; ZLL: Zhanlanlu; ZZY: Zizhuyuan.

## Supplementary Tables

**Supplementary Table 1. The number distribution of the various types of meals with different healthy score and DDS.**

| **Category** | **healthy score** | **DDS** | | | | | | | | | | **N**  **(%)** |
| --- | --- | --- | --- | --- | --- | --- | --- | --- | --- | --- | --- | --- |
|  |  | **1** | **2** | **3** | **4** | **5** | **6** | **7** | **8** | **9** | **10** |  |
| **HLR** | **T** |  | 1 | 15 | 21 | 66 | 102 | 122 | 57 | 18 | 1 | 403 (2.19) |
|  | **0** |  | 1 | 15 | 20 | 54 | 89 | 110 | 52 | 14 | 1 | 356 (1.93) |
|  | **1** |  |  |  | 1 | 9 | 7 | 11 | 5 | 4 |  | 37 (0.2) |
|  | **2** |  |  |  |  | 3 | 6 | 1 |  |  |  | 10 (0.05) |
| **ND** | **T** |  | 10 | 709 | 3331 | 2099 | 1052 | 516 | 104 |  |  | **7821 (42.42)** |
|  | **0** |  | 3 | 504 | 2628 | 1466 | 727 | 383 | 81 |  |  | **5792 (31.42)** |
|  | **1** |  | 7 | 189 | 579 | 509 | 271 | 114 | 22 |  |  | 1691 (9.17) |
|  | **2** |  |  | 16 | 122 | 119 | 51 | 19 | 1 |  |  | 328 (1.78) |
|  | **3** |  |  |  | 2 | 5 | 3 |  |  |  |  | 10 (0.05) |
| **SET** | **T** |  | 3 | 311 | 2362 | 2699 | 1189 | 343 | 93 | 3 |  | **7003 (37.99)** |
|  | **0** |  | 2 | 172 | 1580 | 1453 | 653 | 197 | 79 | 2 |  | **4138 (22.45)** |
|  | **1** |  | 1 | 117 | 599 | 969 | 432 | 129 | 12 | 1 |  | **2260 (12.26)** |
|  | **2** |  |  | 21 | 177 | 244 | 99 | 17 | 2 |  |  | 560 (3.04) |
|  | **3** |  |  | 1 | 6 | 33 | 5 |  |  |  |  | 45 (0.24) |
| **ST** | **T** |  | 12 | 153 | 618 | 540 | 222 | 33 | 1 |  |  | 1579 (8.57) |
|  | **0** |  | 3 | 40 | 314 | 266 | 138 | 17 | 1 |  |  | 779 (4.23) |
|  | **1** |  | 5 | 96 | 215 | 196 | 67 | 16 |  |  |  | 595(3.23) |
|  | **2** |  | 4 | 17 | 89 | 78 | 17 |  |  |  |  | 205(1.11) |
| **WFF** | **T** | 1 | 26 | 87 | 356 | 669 | 433 | 53 | 4 |  |  | 1629 (8.84) |
|  | **0** | 1 | 1 | 60 | 243 | 377 | 152 | 34 | 3 |  |  | 871 (4.72) |
|  | **1** |  | 2 | 11 | 51 | 106 | 54 | 4 |  |  |  | 228 (1.24) |
|  | **2** |  | 23 | 16 | 60 | 182 | 227 | 15 | 1 |  |  | 524 (2.84) |
|  | **3** |  |  |  | 2 | 4 |  |  |  |  |  | 6 (0.03) |
| **N (%)** | | 1  (0.01) | 52  (0.28) | 1275  (6.92) | **6688**  **(36.28)** | **6073**  **(32.94)** | 2998  (16.26) | 1067  (5.79) | 259  (1.4) | 21  (0.11) | 1  (0.01) | 18435 |
| ND: Noodles and dumplings; WFF: Western fast food; SET: Set meal; ST: Staple; HLR: Healthy and light recipes; UN: Unknown. DDS: dietary diversity score; T: total number of meals; | | | | | | | | | | | | |

**Supplementary Table 2. Differences in healthy score (*l*) among various types of meals by Welch ANOVA analysis.**

| Category | N | Mean | SD | Welch *F* | *p* |
| --- | --- | --- | --- | --- | --- |
| HLR | 403 | 0.14 | 0.41 | 260.427 | <0.001** |
| ND | 7821 | 0.30 | 0.55 |  |  |
| SET | 7003 | 0.50 | 0.67 |  |  |
| ST | 1579 | 0.64 | 0.70 |  |  |
| WFF | 1629 | 0.79 | 0.91 |  |  |
| Total | 18435 | 0.45 | 0.67 |  |  |
| ND: Noodles and dumplings; WFF: Western fast food; SET: Set meal; ST: Staple; HLR: Healthy and light recipes; UN: Unknown. * p<0.05 ** p<0.01 | | | | | |

**Supplementary Table 3. Differences in DDS among various types of meals by Welch ANOVA analysis.**

| Category | N | Mean | SD | Welch *F* | *p* |
| --- | --- | --- | --- | --- | --- |
| HLR | 403 | 6.34 | 1.40 | 184.440 | <0.001** |
| ND | 7821 | 4.70 | 1.11 |  |  |
| SET | 7003 | 4.88 | 1.00 |  |  |
| ST | 1579 | 4.58 | 0.95 |  |  |
| WFF | 1629 | 4.96 | 1.00 |  |  |
| Total | 18435 | 4.82 | 1.08 |  |  |
| ND: Noodles and dumplings; WFF: Western fast food; SET: Set meal; ST: Staple; HLR: Healthy and light recipes; UN: Unknown. * p<0.05 ** p<0.01 | | | | | |

**Supplementary Table 4. Differences in standardized TUHII among various types of food outlets within different quantiles of TUHII.**

| Category | Q1 | | | Q2 | | | Q3 | | | Q4 | | | Total | | | Welch  F | p |
| --- | --- | --- | --- | --- | --- | --- | --- | --- | --- | --- | --- | --- | --- | --- | --- | --- | --- |
|  | N | Mean | SD | N | Mean | SD | N | Mean | SD | N | Mean | SD | N | Mean | SD |  |  |
| HLR | 2 | -0.40 | 0.00 | 3 | -0.37 | 0.02 | 6 | -0.28 | 0.08 | 1 | 0.24 | / | 12 | -0.28 | 0.18 | 7.55 | <0.001  ** |
| ND | 93 | -0.39 | 0.00 | 99 | -0.37 | 0.01 | 79 | -0.24 | 0.08 | 71 | 0.76 | 1.03 | **342** | -0.11 | 0.65 |  |  |
| SET | 77 | -0.39 | 0.00 | 66 | -0.37 | 0.01 | 76 | -0.25 | 0.08 | 85 | **1.34** | 2.13 | **304** | **0.13** | 1.35 |  |  |
| ST | 3 | -0.39 | 0.00 | 5 | -0.38 | 0.02 | 12 | -0.22 | 0.10 | 12 | 1.06 | 1.64 | 32 | 0.22 | 1.18 |  |  |
| UN | 24 | -0.39 | 0.00 | 20 | -0.38 | 0.01 | 16 | -0.25 | 0.09 | 5 | 0.33 | 0.32 | 65 | -0.30 | 0.21 |  |  |
| WFF | 4 | -0.39 | 0.00 | 10 | -0.38 | 0.01 | 14 | -0.26 | 0.09 | 29 | **0.80** | 0.93 | 57 | **0.25** | 0.87 |  |  |
| Total | 203 | -0.39 | 0.00 | 203 | -0.37 | 0.01 | 203 | -0.25 | 0.08 | 203 | 1.02 | 1.62 | 812 | 0.00 | 1.00 |  |  |
| ND: Noodles and dumplings; WFF: Western fast food; SET: Set meal; ST: Staple; HLR: Healthy and light recipes; UN: Unknown. * p<0.05 ** p<0.01 | | | | | | | | | | | | | | | | | |

**Supplementary Table 5. Differences in standardized HII among healthy food outlets within different quantiles of HII.**

| Category | Q1 | | | Q2 | | | Q3 | | | Q4 | | | Total | | |
| --- | --- | --- | --- | --- | --- | --- | --- | --- | --- | --- | --- | --- | --- | --- | --- |
|  | N | Mean | SD | N | Mean | SD | N | Mean | SD | N | Mean | SD | N | Mean | SD |
| HLR |  |  |  |  |  |  |  |  |  | 2 | 2.92 | 0.97 | 2 | 2.92 | 0.97 |
| ND | 11 | -0.56 | 0.01 | 15 | -0.51 | 0.02 | 17 | -0.32 | 0.11 | 13 | 0.97 | 1.33 | **56** | -0.12 | 0.87 |
| SET | 16 | -0.56 | 0.01 | 8 | -0.50 | 0.03 | 10 | -0.35 | 0.08 | 14 | 1.63 | 0.94 | **48** | 0.13 | 1.09 |
| ST |  |  |  |  |  |  |  |  |  | 1 | 0.88 | / | 1 | 0.88 | / |
| UN | 4 | -0.56 | 0.00 | 6 | -0.50 | 0.03 | 4 | -0.31 | 0.05 | 1 | 0.34 | / | 15 | -0.41 | 0.23 |
| WFF |  |  |  | 1 | -0.54 | / |  |  |  |  |  |  | 1 | -0.54 | / |
| Total | 31 | -0.56 | 0.01 | 30 | -0.51 | 0.02 | 31 | -0.32 | 0.09 | 31 | 1.37 | 1.19 | 123 | 0.00 | 1.00 |
| ND: Noodles and dumplings; WFF: Western fast food; SET: Set meal; ST: Staple; HLR: Healthy and light recipes; UN: Unknown. | | | | | | | | | | | | | | | |

**Supplementary Table 6. Differences in standardized UHII among unhealthy food outlets within different quantiles of UHII.**

| Category | Q1 | | | Q2 | | | Q3 | | | Q4 | | | Total | | | Welch F | p |
| --- | --- | --- | --- | --- | --- | --- | --- | --- | --- | --- | --- | --- | --- | --- | --- | --- | --- |
|  | N | Mean | SD | N | Mean | SD | N | Mean | SD | N | Mean | SD | N | Mean | SD |  |  |
| HLR |  |  |  | 3 | -0.51 | 0.07 | 5 | -0.15 | 0.23 | 2 | 0.68 | 0.52 | 10 | -0.09 | 0.50 | 6.48 | <0.001  ** |
| ND | 65 | -0.72 | 0.02 | 68 | -0.55 | 0.08 | 66 | -0.14 | 0.17 | 87 | 1.38 | 1.06 | **286** | 0.09 | 1.06 |  |  |
| SET | 63 | -0.71 | 0.02 | 59 | -0.56 | 0.08 | 69 | -0.12 | 0.18 | 65 | 1.43 | 1.14 | **256** | 0.02 | 1.03 |  |  |
| ST | 8 | -0.71 | 0.02 | 8 | -0.55 | 0.07 | 7 | -0.06 | 0.17 | 8 | 1.83 | 1.52 | 31 | 0.13 | 1.28 |  |  |
| UN | 22 | -0.71 | 0.02 | 18 | -0.56 | 0.09 | 6 | -0.17 | 0.20 | 4 | 1.60 | 0.88 | 50 | -0.41 | 0.66 |  |  |
| WFF | 14 | -0.71 | 0.02 | 16 | -0.57 | 0.08 | 19 | -0.05 | 0.20 | 7 | 0.61 | 0.33 | 56 | -0.28 | 0.47 |  |  |
| Total | 172 | -0.71 | 0.02 | 172 | -0.56 | 0.08 | 172 | -0.12 | 0.18 | 173 | 1.38 | 1.09 | 689 | 0.00 | 1.00 |  |  |
| ND: Noodles and dumplings; WFF: Western fast food; SET: Set meal; ST: Staple; HLR: Healthy and light recipes; UN: Unknown. * p<0.05 ** p<0.01 | | | | | | | | | | | | | | | | | |

**Supplementary Table 7. Differences in health weight among unhealthy food outlets within different quantiles of health weight.**

| Category | H | | | rH | | | ruH | | | uH | | | Total | | | Welch F | p |
| --- | --- | --- | --- | --- | --- | --- | --- | --- | --- | --- | --- | --- | --- | --- | --- | --- | --- |
|  | N | Mean | SD | N | Mean | SD | N | Mean | SD | N | Mean | SD | N | Mean | SD |  |  |
| HLR | 7 | 0.93 | 0.02 | 2 | 0.78 | 0.04 |  |  |  | 1 | 0.38 | / | 10 | 0.85 | 0.18 | 17.95 | <0.001  ** |
| ND | 99 | 0.90 | 0.04 | 77 | 0.77 | 0.05 | 72 | 0.58 | 0.07 | 38 | 0.23 | 0.16 | 286 | 0.69 | 0.23 |  |  |
| SET | 48 | 0.91 | 0.03 | 54 | 0.76 | 0.05 | 67 | 0.56 | 0.08 | 87 | 0.23 | 0.14 | 256 | 0.55 | 0.28 |  |  |
| ST | 2 | 0.93 | 0.02 | 9 | 0.75 | 0.05 | 6 | 0.56 | 0.08 | 14 | 0.15 | 0.12 | 31 | 0.46 | 0.31 |  |  |
| UN | 13 | 0.88 | 0.02 | 15 | 0.79 | 0.04 | 19 | 0.55 | 0.07 | 3 | 0.29 | 0.04 | 50 | 0.69 | 0.18 |  |  |
| WFF | 4 | 0.90 | 0.05 | 15 | 0.76 | 0.05 | 8 | 0.61 | 0.05 | 29 | 0.12 | 0.13 | 56 | 0.42 | 0.33 |  |  |
| Total | 173 | 0.90 | 0.04 | 172 | 0.77 | 0.05 | 172 | 0.57 | 0.07 | 172 | 0.21 | 0.15 | 689 | 0.61 | 0.28 |  |  |
| ND: Noodles and dumplings; WFF: Western fast food; SET: Set meal; ST: Staple; HLR: Healthy and light recipes; UN: Unknown. H: healthy; rH: relatively healthy; ruH: relatively unhealthy; uH: unhealthy. * p<0.05 ** p<0.01 | | | | | | | | | | | | | | | | | |

# Appendix 1

The rules to calculate the category of food outlets from the category of the meals are as follows:

First, calculate the basic parameters used in next category calculation, including the following: the number of every type of meal in each food outlet (N_m_); the maximum, minimum, standard deviation (STD); the ratio of the maximum and minimum (MMR) of N_m_; the standard deviation of two meals with the largest number (STD-FT); and the difference between STD and STD-FT (D-STD).

Second, the type of food outlet was determined by the following rules:

1. When STD was non-numerical, it indicated that the food outlet sold only one type of meal; therefore, the type of the meal was directly set as the type of the food outlet.

2. When STD was numerical, it was discussed as follows:

1) When the maximum of all types of meals were less than or equal to 10, it indicated that there may be omissions of meals sold in this food outlet, or the main offline business of the store was better than the online business, or other unknown reasons led to the low number of each type of meals in the food outlet. Therefore, this type of food outlet was temporarily classified as unknown.

2) When the maximum of all types of meals were more than 10, there were two situations:

A. If D-STD was more than 0, the food outlet was classified as unknown;

B. If D-STD was less than or equal to 0, it depended on the following two conditions:

a. If MMR was more than 3, the food outlet was classified as the type of meal corresponding to the maximum of meal;

b. If MMR was less than or equal to 3, the food outlet was classified as unknown.

References

Briggs, D., Corvalán, C., Nurminen, M., 1996. Linkage Methods for Environment and Health Analysis: General Guidelines. UNEP, USEPA, WHO, Geneva, Switzerland, 148 pp. https://www.emerald.com/insight/content/doi/10.1108/MEQ-11-2012-0075/full/pdf?title=environmental-health-indicators-a-review-of-initiatives-worldwide.
